# A Tandem Ring Closure and Nitrobenzene Reduction with Sulfide Provides an Improved Route to an Important Intermediate for the Anti-Tuberculosis Drug Candidate Sutezolid

**DOI:** 10.1021/acs.oprd.4c00014

**Published:** 2024-03-27

**Authors:** Hanuman P. Kalmode, Ongolu Ravikumar, Dinesh J. Paymode, John Bachert, Justina M. Burns, Rodger W. Stringham, Sarah L. Aleshire, Ryan C. Nelson

**Affiliations:** Medicines for All Institute, 737 N Fifth St., Box 980100, Richmond, Virginia 23298, United States

**Keywords:** tuberculosis, sutezolid, thiomorpholine, Zinin reduction

## Abstract

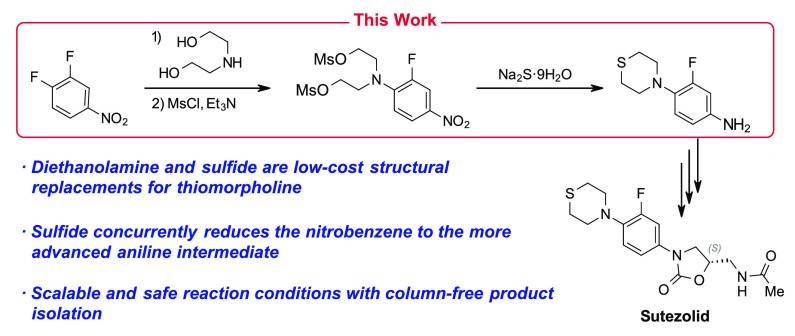

Sutezolid is an in-development thiomorpholine derivative of the FDA-approved tuberculosis (TB) treatment linezolid. Current synthetic routes for preparing sutezolid start with thiomorpholine as a key structural building block; unfortunately, this material was identified as a major cost driver for the API, which will limit the potential uptake of this treatment in lower income regions. In this work, an alternative, lower-cost synthetic strategy to a known *p*-phenylenediamine intermediate to sutezolid has been demonstrated. The key step in this process is the construction of the thiomorpholine ring by a nucleophilic sulfide ring closure on an activated bis(2-hydroxyethyl)-functionalized aniline, which was in turn made by reaction of 3,4-difluoronitrobenzene and diethanolamine. This sulfide treatment has the added benefit of affecting a Zinin reduction of the nitro functional group, which alleviates the need for the transition metal reduction used in previous routes. After optimization, this key reaction was able to provide the desired aniline intermediate in yields between 65 and 80% and, after a standard charcoal treatment, purity of >94%. Initial demonstrations of the full 3-step strategy were successfully conducted on scales up to 100 g with overall yields of 53–68%. This preliminary work will serve as the foundation for a broader low-cost redesign of the sutezolid synthetic process.

## Introduction

Tuberculosis (TB) remains one of the deadliest infectious diseases in the world, with nearly 11 million cases worldwide in 2021 accounting for 1.6 million deaths. These sobering statistics are made more upsetting by the fact that variety of treatments for TB are available, and in cases where adequate treatments are provided in a timely manner, TB infections can be curable.^1,2^ Unfortunately, the majority of TB cases occur in low-to-middle income countries (LMIC), where access to low-cost, effective medications can be limited. As an additional complication, a lack of treatment options can give rise to multidrug-resistant TB strains (MDR-TB).^3^ After a decades-long drought of new TB treatments,^4^ several new drugs have been developed in recent years, including bedaquiline,^5,6^ linezolid, pretomanid,^7^ and delamanid,^8^ and combination regimens of these drugs such as BPaL.^9,10^ However, ensuring low costs for these new treatment options remains a top priority for the global health community.

**Chart 1:**
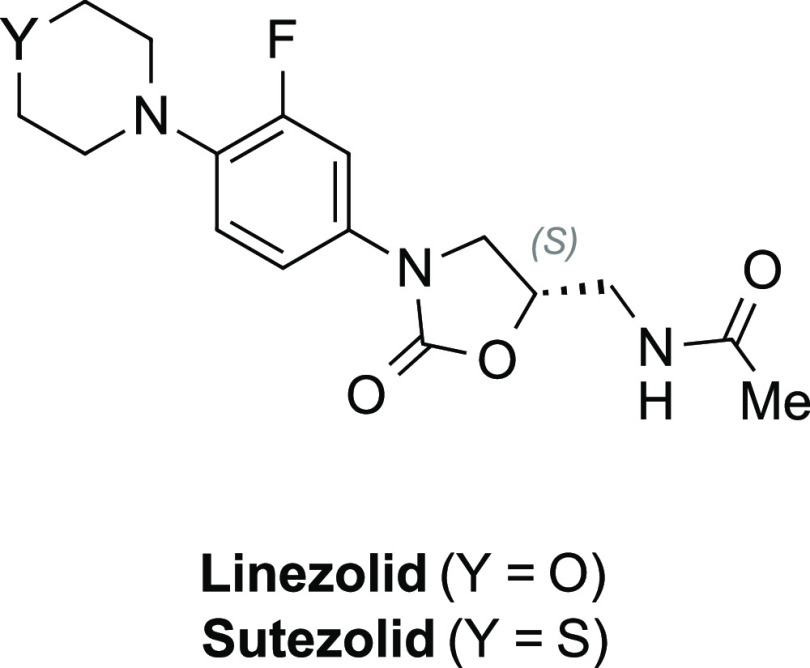
Linezolid and Sutezolid, Two Oxazolidinone-Based Antibiotics

Linezolid (Chart 1), a member of the oxazolidinone class of antibiotics, was approved by the FDA in 2000 as a treatment for drug-resistant strains of TB. Unfortunately, with prolonged treatment, some patients experience toxic side effects, which limits linezolid’s broader acceptance.^11^ Over the years, a number of linezolid variants have been synthesized in an effort to reduce adverse effects while potentially improving efficacy. From this effort, it was discovered that the thiomorphline-substituted analog sutezolid appears to meet both of these goals.^12^ This drug is being developed by group of organizations coordinated by the TB Alliance, who have taken it into Phase 2 clinical trials.^13^ As sutezolid makes its way through the remaining trials, a low-cost synthetic route to this compound will be a crucial factor in ensuring that treatments containing this potential therapy are affordable options for the LMIC markets.

**Scheme 1:**
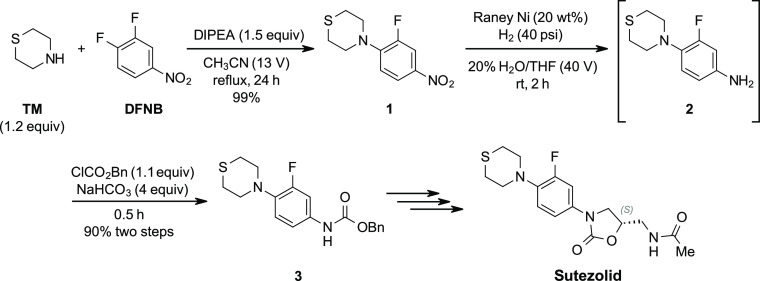
Early Steps in a Typical Synthetic Route to Sutezolid^12^

Initial synthetic routes toward sutezolid made use of the well-developed chemistry for its cousin linezolid (Scheme 1).^12,14−17^ In these routes, an S_N_Ar coupling of thiomorpholine (TM) and 3,4-difluoronitrobenzene (DFNB) forms nitrobenzene intermediate **1**. This is followed by a reduction of the nitro group to aniline **2**, which can be accomplished by using either catalytic hydrogenation or stoichiometric transition metals. This aniline is then capped and isolated as a carbamate (**3**). Completion of the oxazolidinone ring can be accomplished by several methods that make use of chiral C3 fragments derived from either glycidol or epichlorohydrin. Although the early steps in this route are seemingly simple, a technoeconomic analysis of this chemistry identified TM as the most expensive structural component of this molecule by a wide margin. Incorporation of this fragment early in the synthetic route further inflates its overall cost contribution, rendering these early routes impractical from a cost perspective. As a second concern, transition metal reductions can introduce some issues that may affect cost, such as the complete removal of trace metals from the product or the need for pressurized systems under catalytic conditions. The primary goals then for a lower-cost synthetic route were identifying a low-cost thiomorpholine alternative and, if possible, avoiding the transition metal reduction.

A collaborative team of the University of Graz and the Medicines for All Institute validated a low-cost synthesis of TM using an efficient thiol-ene/cylization reaction sequence between vinyl chloride and cysteamine.^18^ Because this chemistry has the potential to generate mustard-like intermediates, safety was a primary concern, so the process was transitioned to a continuous flow apparatus to limit manipulation of potentially hazardous intermediates. Even with this novel synthetic procedure in hand, alternative routes to TM or other TM-containing intermediates were still desired to expand the potential manufacturing base for intermediates or the API.

**Scheme 2:**
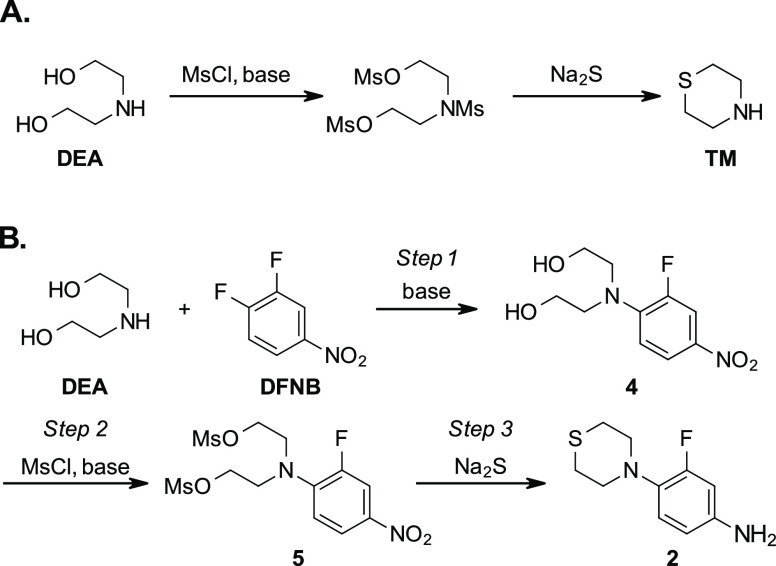
(A) Known Synthesis of Thiomorpholine That Inspired (B) a Proposed Route to a Known Thiomorpholinylaniline Intermediate (**2**) to Sutezolid

With the goal of providing synthetic alternatives to the known TM-based routes to sutezolid, the literature preparations for TM were reevaluated with an eye for potential modifications that would avoid the direct synthesis and isolation of TM. One early synthetic report in particular described a ring-closing reaction between diethanolamine (DEA) and sodium sulfide, making use of mesylate for hydroxyl activation (Scheme 2A).^19^ This chemistry suggested that a similar sulfide treatment of dimesyl intermediate **5** may provide an attractive alternative route to a known intermediate sutezolid (Scheme 2B). A major advantage of this chemistry is that DEA and sodium sulfide are low-cost raw materials, which make them ideal structural building blocks. In addition, the coupling reaction between DFNB and DEA to form the bis(2-hydroxyethyl)-functionalized aniline (**4**) is known,^20^ and by closing the ring after connecting these two fragments, the isolation of TM can be avoided. Unlike other cyclization reactions to make TM, mesylate-activated intermediate **5** in this potential route should be less prone to unwanted intramolecular reactions because of the poor nucleophilicity of the aniline. Finally, the treatment of **5** with sulfide has the potential to affect two chemical transformations: the closure of the thiomoropholinyl ring and a Zinin reduction^21^ of the nitrobenzene to the known aniline intermediate **2**. If successful, this sequence would then meet both design criteria: avoiding the most expensive structural component and transition metal reduction. In this manuscript, we will present a feasibility assessment of this route, which represents the first step in our larger route redesign project.^22^

## Results and Discussion

### Step 1: Condition Screening for the S_N_Ar Reaction

The S_N_Ar reaction between DEA and DFNB is robust and tolerates a wide variety of conditions (Table 1). Reactions conducted with near stoichiometric DEA and trialkylamine bases in CH_3_CN (entries 1–4) show clean product formation, but they are sluggish, even at temperatures up to 80 °C. With K_2_CO_3_ or DBU (entries 6 and 7), complete consumption of the starting material is achieved; however, the product mixture contains a significant portion of a single impurity, which was identified as the double substitution product **6**.

**Table 1:**
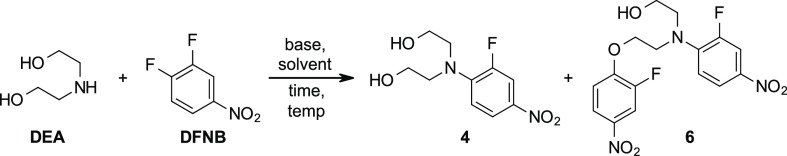
Optimization of the S_N_Ar Reaction between DFNB with DEA^a^

						**HPLC area%**^b^		
**entry**	**DEA (equiv)**	**base (equiv)**	**solvent (V)**	**temp. (°C)**	**time (h)**	**DFNB**	**4**	**6**
1	1.2	Et_3_N (1.5)	CH_3_CN (10)	20	24	83	17	
2	1.2	Et_3_N (1.5)	CH_3_CN (10)	50	24	15	85	
3	1.2	Et_3_N (1.5)	CH_3_CN (10)	80	24	4.6	91	1.5
4	1.2	DIPEA (1.5)	CH_3_CN (10)	80	24	8	92	
5	1.2	K_2_CO_3_ (1.5)	CH_3_CN (10)	80	24		47	42
6	1.2	DBU (1.5)	CH_3_CN (10)	80	24		69	22
7	3.0		CH_3_CN (5)	80	3	2.9	95.4	1.7
8	3.0		Toluene (5)	100	3	1.7	94.2	4
9	3.0		DMF (5)	100	3		92	1.5
10	3.0		H_2_O (5)	100	3	1.8	91.8	6.4
**11**	**3.0**			**80**	**3**		**96.5**	**3.5**
12	3.0			50	24		96	4
13	3.0			20	24	27.5	69	3.5
14	2.0			90	3		93	7
15	5.0			90	3		96.5	3.5

a Reaction conditions: DFNB (200 mg), DEA, base, and solvent were combined in a small vial. This was heated to the given temperature, and after the indicated time, a sample was taken directly from the crude reaction mixture for HPLC analysis.

b Data collected at 210 nm.

Given the similarity in both basicity and large-scale availability of DEA and Et_3_N, a series of reactions using DEA as both the reactant and base were explored (Table 1, entries 7–10). In reduced volumes of CH_3_CN (5 V), near complete conversion was obtained with 3 equiv of DEA in only 3 h at 80 °C (entry 7). Toluene, DMF, and water were also assessed as solvents (entries 8–10), but conversion to product in all of these cases was lower than that in the CH_3_CN system. However, in water, the product precipitates as a yellow solid upon cooling to room temperature, which suggests that water would make a suitable antisolvent for column-free isolation at scale.

At slightly elevated temperatures, DEA is a free-flowing liquid, so it was postulated that additional rate and throughput improvements could be achieved under neat conditions with DEA acting as the reaction solvent as well (Table 1, entries 11–15). In a neat reaction of DFNB and DEA (3 equiv) at 80 °C, complete consumption of the starting material is observed along with a small amount of impurity **6** (entry 11). Lowering the reaction temperature does not reduce the relative amount of **6** (entries 12 and 13), and at these temperatures, the mixture becomes considerably more viscous, reducing the reaction rate and limiting efficient stirring. The reaction remains effective even at the stoichiometric limit of DEA (entry 14), but at the expense of elevated impurity levels. In contrast, increasing the amount of DEA (5 equiv) reduces the impurity level due to the overall reduction in concentration; however, the excess reagent may be more challenging to remove at the end of the reaction. With this in mind, a slight excess of DEA (3 equiv) and temperatures between 80 and 90 °C were chosen for further scale-up optimization.

### Step 1: S_N_Ar Reaction Safety

While the S_N_Ar reaction was transitioned to multigram scales, a substantial exotherm was observed, which prompted a safety assessment to ensure scalable conditions were possible. When DEA (3 equiv) and DFNB are mixed at room temperature and then heated, a rapid increase in the internal temperature is observed starting at ∼50–60 °C. The highly exothermic nature of this process can be explained by a combination of the acid/base neutralization of HF with DEA and the formation of an extensive hydrogen bond network between fluoride and the OH and NH donors. Dilution of the reaction mixture with CH_3_CN was considered to buffer the heat release; however, because of the lack of reactivity at lower temperatures, the reaction would still require external heating to temperatures close to the boiling point of the solvent. In that case, an unexpected exothermic event may cause evaporation of the solvent. Given the much higher boiling point of DEA, the use of this material as the solvent was maintained, but an alternative mode of addition was explored to ensure a facile, but also safe, reaction.

**Scheme 3:**
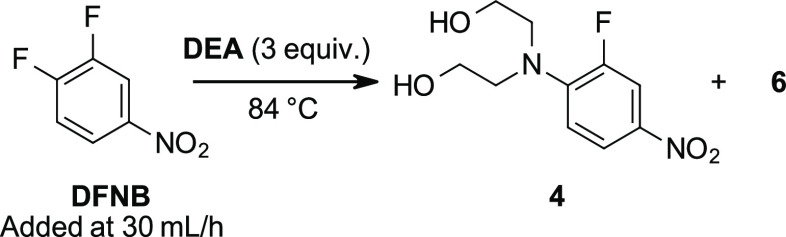
Slow-Addition Conditions for the S_N_Ar Reaction between DFNB and DEA

**Figure 1:**
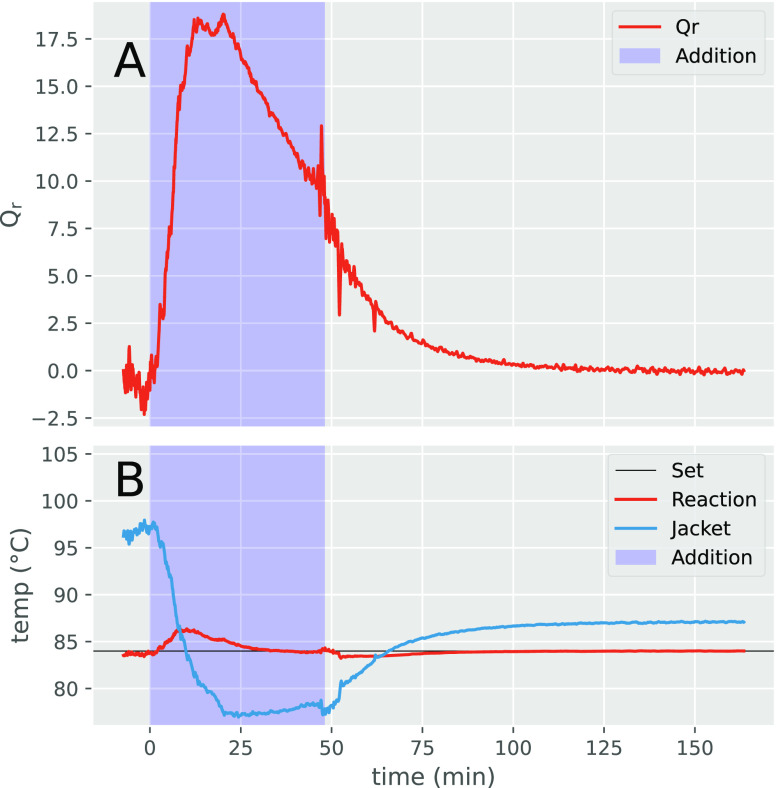
(A) Heat flow and (B) reaction and jacket temperatures measured during the slow addition (blue shaded area) of DFNB to DEA at 80 °C.

A common method employed to control exothermic reactions that require elevated temperatures is the slow addition of the reactive reagent to the hot reaction mixture, adjusting the addition rate to ensure manageable heat dissipation. This methodology was tested by conducting a slow addition of DFNB (35.0 g) to hot DEA (3.0 equiv., Scheme 3) using an EasyMax reactor to measure the heat flow of reaction (*Q*_r_, Figure 1), while the reaction progress was assessed by periodic HPLC measurements (Table 2). In this experiment, DFNB was added at a rate of 30 mL/h via a syringe pump to DEA held at 84 °C. Immediately upon addition, an exothermic spike was observed (Figure 1A) along with a modest increase in the reaction temperature (Figure 1B, red line), the latter of which was modulated by a reduction in the external reactor temperature (blue line). After roughly 10 min, the heat flow reached a short plateau, and an IPC taken at this point shows a product-to-starting material ratio of 2.5:1, with only a small amount impurity **6** detected (entry 1). The relative ratio of product to starting material remained roughly unchanged throughout the remainder of the addition (entries 2–3), which suggests DFNB is being steadily consumed at this addition rate without a buildup of reactive species. After 2 h of total reaction time, complete conversion of the starting material was achieved (entry 4). To maintain safe conditions for subsequent reactions, the addition rate of DFNB was controlled such that the internal reaction temperature did not exceed the set temperature by more than ∼5 °C.

**Table 2:** In-Process Control Data Taken During the Slow-Addition Experiment

		**HPLC area%**^a^		
**entry**	**time (min)**	**DFNB**	**4**	**6**
1	9	28.6	71.1	2.2
2	30	20.2	75.4	3.5
3	50	20.6	72	5.9
4	110	0	93.8	5.8

a Data collected at 210 nm.

### Step 1: S_N_Ar Reaction Scale-Up, Isolation, and Impurity Identification

With optimized reaction conditions and a safe mode of addition in hand, the substitution reaction was conducted several times on scales up to 100 g (Table 3). Upon completion of the reaction, precipitation of the crude yellow product was achieved by controlled addition of water (5 V) to the hot reaction mixture followed by cooling to RT. This crude product was isolated by filtration, and an MTBE wash (2 V) was found to reduce the dimer impurity to ∼2.5–3 area%. At this level, the remaining dimer impurity was purged in subsequent steps. After drying overnight, the product was isolated in high yields (91–95%) and good assay purity (97%).

**Table 3:**
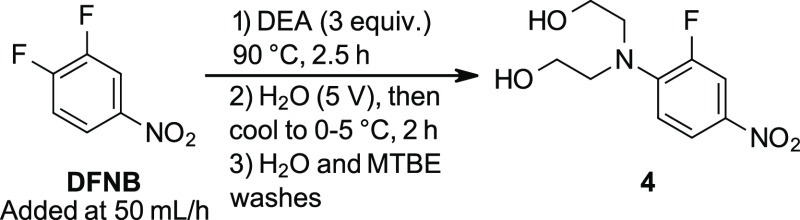
Results of the Scaled S_N_Ar Coupling of DFNB and DEA

**entry**	**scale (g)**	**temp. (°C)**	**time (h)**	**LCAP**^a^	**assay (%)**^b^	**yield (%)**
1	30	80	3.0	97.3	97.4	91.4
2	50	90	2.5	96.7	97.2	94.3
3	100	90	2.5	96.8	97.4	94.8

a Data collected at 210 nm.

b Assay determined using a standard of 4 with a known purity of 97.99%.

### Step 2: Mesylation Reaction Screening and Optimization

A series of common conditions were screened to determine the optimal base and solvent combinations for the mesylation of **4** (Table 4). Reactions with inorganic bases and imidazole were sluggish after several hours at RT, and heating to reflux overnight did little to improve conversion (entries 1–5). On the other hand, Et_3_N afforded full conversion to the desired product in both CH_3_CN and DCM without the need for external heating. In fact, IPC data taken from the CH_3_CN reaction showed that nearly full conversion was achieved after only a few hours, and internal temperature measurements taken during the course of the reaction did not point to a substantial exotherm for this process.

**Table 4:**
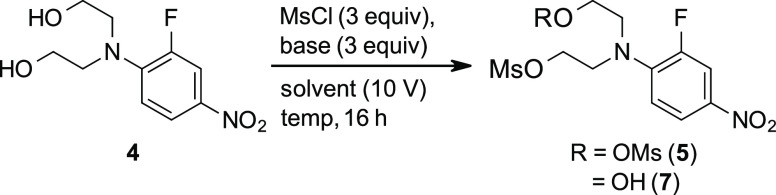
Screening Conditions for the Mesylation of **4**^a^

				**HPLC area%**^b^		
**entry**	**base**	**solvent**	**temp. (°C)**	**4**	**5**	**7**
1	K_2_CO_3_	acetone	0 to reflux	20	27	53
2	Na_2_CO_3_	Acetone	0 to reflux	20	27	53
3	KO^*t*^Bu	THF	0 to reflux	14	35	25
4	NaO^*t*^Bu	CH_3_CN	0 to reflux	10	66	23
5	Imidazole	CH_3_CN	0 to reflux	74	6	18
6	Et_3_N	CH_3_CN	0 to 20		100	
7	Et_3_N	CH_2_Cl_2_	0 to 20		100	

a Reaction Conditions: **4** (200 mg), 3.0 equiv of MsCl, 3.0 equiv of base, and 10 V of solvent were combined in a small vial. This was heated to the given temperature, and after 16 h, a sample was taken directly from the crude reaction mixture for HPLC analysis.

b HPLC data collected on crude reaction mixture, measured at 380 nm.

**Figure 2:**
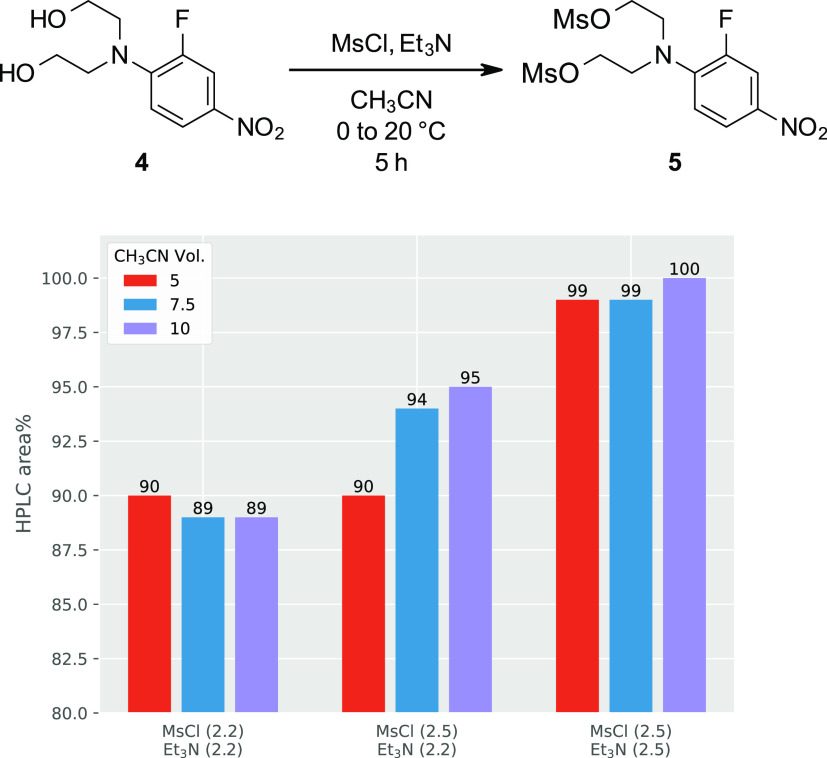
Probing the effect of MsCl and Et_3_N equivalents and solvent volumes on the efficiency of the mesylation of **4**. HPLC data were collected from the crude reaction mixture at 380 nm.

A second series of screening reactions was conducted to further optimize reagent and solvent utilization (Figure 2). With a slight excess of both Et_3_N and MsCl (2.2 equiv each), incomplete conversion was observed at solvent loadings ranging from 5 to 10 V. With elevated equivalents of MsCl (2.5 equiv), higher conversions were achieved but only in the most dilute conditions (10 V). However, with 2.5 equiv of both MsCl and Et_3_N, complete conversion was possible at solvent levels as low as 5 V, so these conditions were chosen for further scaling.

With the optimized reaction conditions in hand, the scalability of this process could be assessed. As with **4**, the dimesylate **5** precipitates upon addition of water to the reaction mixture, providing a chromatography-free isolation method. To optimize the ratio of solvent to antisolvent, a series of reactions on a 30 g scale with varying solvent and antisolvent volumes were conducted (Table 5, entries 1–4). In all cases, solid precipitation was observed upon addition of water at room temperature; however, the filtrations and washes were conducted at 0–5 °C to ensure minimal product loss in the mother liquor. With the smallest charge of antisolvent (entry 1), the product was isolated in excellent purity (100%) but modest yield (82.9%). Isolated yields improved with equal volume ratios of CH_3_CN to water (entries 3 and 4); however, the reaction mass under these conditions became sticky, making filtration difficult. A compromise between isolated yield and filterability was achieved with an intermediate addition of water (3.5 V, entry 2), where the product was isolated in good yield and purity (85.2 and 99.6%, respectively). The mesylation reaction was further demonstrated at scales up to 100 g (entries 5 and 6). At these scales, the isolated yields improved to 90% with assay purities of ≥98% (compared to a standard with purity >99%), which was sufficient to proceed into the final stage.

**Table 5:**
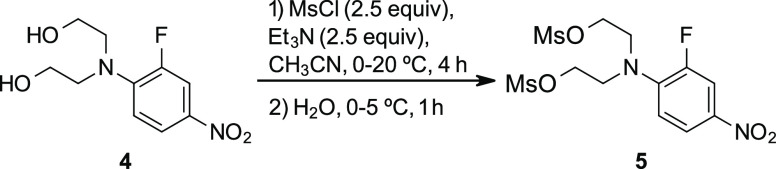
Assessing Product Recovery and Purity on the Basis of Solvent and Antisolvent Volumes

**entry**	**scale (g)**	**MeCN (V)**	**water (V)**	**assay (%)**^a^	**yield (%)**
1	30	5.0	2.5	100.1	82.9
2	30	5.0	3.5	99.6	85.2
3	30	5.0	5.0	99.6	89.5
4	30	7.5	7.5	101.2	89.0
5	50	5.0	4.0	98.9	90.3
6	100	5.0	4.0	99.2	89.8

a HPLC data measured at 380 nm using a standard with a purity >99%.

### Step 3: Ring Closure and Reduction with Sodium Sulfide

The key reaction in this route redesign, the tandem ring closure and nitro reduction with sulfide, was assessed in a variety of solvents (Table 6, entries 1–5). Except for water, where no reaction occurred, the desired product **2** was observed in all solvents. However, at least three additional species were present in the crude mixtures: partially reacted **1**, morpholinylaniline **8**, and unidentified component **9**. Although water on its own was not a suitable solvent, previous kinetic studies on the Zinin reduction suggest that water is important to ensure high solubility of reactants and to maintain a highly alkaline reaction medium, which favors the more reductive deprotonated sulfide species.^21^ To balance these competing needs, several reactions were attempted in mixtures of water and organic solvents. Mixtures of water and THF or CH_3_CN (entries 6 and 7) did not show improvement over their nonaqueous counterparts; however, in a 3:2 mixture of water and EtOH, full conversion was achieved in only 3 h (entry 8) with the desired compound **2** being the major component as assessed by HPLC (89 area%). Any change in the relative concentration of ethanol reduced the conversion to aniline (entries 9 and 10), as did lowering either the equivalents of sulfide or the reaction temperature (entries 11–15). Although the best conditions (entry 8) were highly selective for **2**, the impurities **8** and **9** were present at 2.5 and 8.5 area%, respectively, so an effort to understand the formation of these compounds was undertaken with the goal of developing suitable methods to reduce these unwanted products.

**Table 6:**
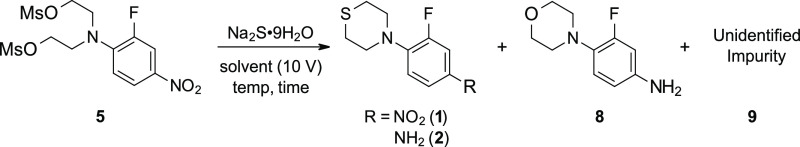
Screening Conditions for the Tandem Ring Closure/Reduction of **5** with Na_2_S·9H_2_O^a^

					**HPLC area%**^b^				
**entry**	**Na**_**2**_S·9H_**2**_**O(equiv)**	**solvent**	**temp. (°C)**	**tme (h)**	5	1	2	8	9
1	3.0	CH_3_CN	50	24		30	64	2.6	2.7
2	3.0	EtOH	50	48			83		17
3	3.0	THF	50	48		12	84		4
4	3.0	H_2_O	50	48	100				
5	3.0	*^i^*PrOAc	50	48		43	4.5		3
6	3.0	THF/H_2_O (3:2)	50	48	100				
7	3.0	CH_3_CN/H_2_O (3:2)	80	24		24	67	4	4
**8**	**3.0**	**EtOH/H**_**2**_**O**(3:2)	**80**	**3**			**89**	**2.5**	**8.5**
9	3.0	EtOH/H_2_O (1:4)	80	3		9	80	5	5
10	3.0	EtOH/H_2_O (4:1)	80	3			87.5	2.5	10
11	2.0	EtOH/H_2_O (3:2)	20	96	46.6	13.7	30	0.8	4.7
12	2.0	EtOH/H_2_O (3:2)	80	96		29.7	66	1.2	3
13	2.5	EtOH/H_2_O (3:2)	20	96		15.6	74	0.9	9
14	2.5	EtOH/H_2_O (3:2)	80	96		14.5	79.8	2.7	2.9
15	3.0	EtOH/H_2_O (3:2)	20	96		10.5	74.5	0.8	11.2

a Reaction Conditions: **5** (200 mg), Na_2_S·9H_2_O, and solvent (10 V) were mixed in a small vial and heated to the given temperature and time.

b HPLC data collected on a small sample of the crude reaction mixture, with detection at 210 nm.

**Figure 3:**
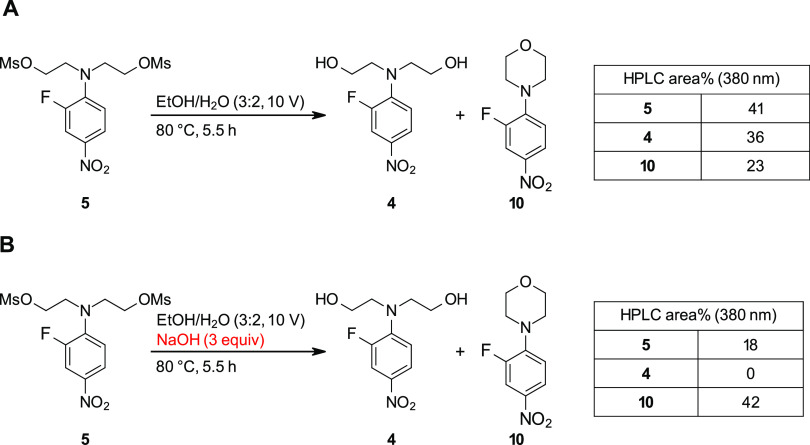
Control reactions for testing the stability of **5** in (A) a heated reaction solvent system and (B) with added base.

The morpholinylaniline derivative **8** is likely formed by an initial hydrolysis of a mesylate followed by a base-facilitated ring closure, with both steps being accelerated by the alkaline reaction conditions. To confirm this hypothesis, two control reactions were conducted that involved heating **5** to 80 °C in a 3:2 mixture of ethanol and water (10 V) for 5.5 h. With no additional reagents (Figure 3A), this reaction produced a mixture of morpholinylnitrobenzene **10**, fully hydrolyzed **4**, and unreacted **5** in a 23:36:41 ratio as determined by HPLC. However, with addition of NaOH (Figure 3B), a substantial increase in **10** is observed (41 area% at 380 nm), along with several unidentified products. These results suggest that basicity needs to be limited during the ring-closing reaction to ensure the reaction with sulfide rather than hydroxide. This conclusion conflicts with the need for alkaline conditions for the nitrobenzene reduction; however, the Zinin reduction has the potential to generate a variety of nucleophilic intermediates that could be the source of the impurity **9**.^23^ Commercial sodium sulfide can also be contaminated with small amounts of polysulfide impurities (e.g., S_2_^2–^),^24^ which could also act as nucleophiles. Taking these considerations into account, it seemed evident that some modifications to the procedure would be necessary to ensure clean reactions in this step.

To facilitate a selective ring closure with sulfide, slow addition of the first equivalent of sulfide was attempted. This slow addition should mediate the pH increase, reducing the morpholinyl formation while also limiting the amount of Zinin-related nucleophilic intermediates. After a short reaction delay, additional sulfide equivalents could be added in one lot to complete the nitro reduction reaction. An initial attempt at this procedure (Table 7, entry 1) showed a dramatic improvement in the conversion to the desired product (96.5 area%), with a large drop in the level of the impurity **9**. This suggests that **9** is a result of oxidized, nucleophilic sulfur species that are either inherently present in the reagent or generated during the Zinin reaction.

**Table 7:**
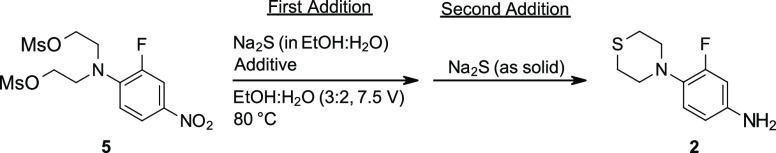
Attempts to Minimize Impurities via a Two-Step Sulfide Addition Sequence

		**first addition**				**HPLC (area%)**^b^		
**entry**	**additive (equiv)**	**Na**_**2**_**S (equiv)**	**addition time (h)**	**total time (h)**	**solid Na**_**2**_**S (eq)**	**2**	**8**	**9**
1	none	1.05	2	4	1.95	96.5	2.7	0.7
2	Na_2_SO_3_ (0.5)	1.2	2	4	1.8	96.3	3.65	0
3	Na_2_SO_3_ (0.5)	1.2	0.5	1	1.8	97.6	2.45	0

a Reaction Conditions: dimesylate **5** (2.0 g, 1 equiv) and the additive are added to a mixture of EtOH and H_2_O (3:2, 15 mL, 7.5 V) at room temperature before being heated to 80 °C. At this point, a solution of Na_2_S·9H_2_O dissolved in EtOH:H_2_O (3:2, 5 mL) was added via a syringe pump over the given addition time. This reaction mixture was held at temperature for the given total time before the second addition of solid Na_2_S·9H_2_O was added. The reaction was left at temperature for an additional 3 h. At the end of the reaction time, the reaction mixture was cooled to room temperature, and a sample of the crude reaction mixture was taken for HPLC analysis.

b Collected at 210 nm.

To limit the unwanted reaction with these putative oxidized sulfur species, the addition of a small amount of reducing agent was considered for the ring closure reaction. In a second two-step addition experiment (Table 7, entry 2), a substoichiometric charge of Na_2_SO_3_ (0.5 equiv), a known disulfide reductant,^25^ included before the start of sulfide addition was found to eliminate the remaining traces of impurity **9**. Unfortunately, this reaction modification also caused a slight increase in the relative level of the morpholine impurity. This was ascribed to the increase in basicity of the reaction mixture with the added Na_2_SO_3_, so the initial Na_2_S addition and reaction times were accelerated to limit the exposure of **5** to the alkaline reaction conditions. With shortened sulfide addition and initial reaction times (entry 3), the level of morpholine impurity (**10**) was reduced back to the original levels, while also ensuring the absence of the putative polysulfide impurity. With these optimized conditions in hand, the scalability of this reaction system could be assessed.

The optimized two-step sulfide addition conditions were conducted several times on scales up to 100 g (Table 8). For product isolation, the reaction mixture was acidified to a pH of approximately 10 and extracted with iPrOAc. The reproducibility at these scales was good. Morpholine impurity (**10**) levels in the isolated product were between 3 and 4% by HPLC at 210 nm, and the unidentified impurity was not detected in any of the samples. Excellent isolated masses were obtained under these conditions; unfortunately, the purity was low, presumably due to inorganic impurities. Several attempts were made to crystallize the product; however, these efforts proved futile due to the amorphous nature of the aniline, which may explain why this compound has typically been used with little to no purification.

**Table 8:**
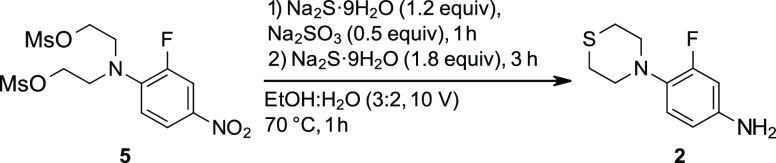
Results of Scaled Two-Step Sulfide Addition Reactions

		**HPLC (area%)**^a^				
**entry**	**sale (g)**	**2**	**8**	**crude mass (g)**	**assay (%)**	**yield (%)**
1	20	96.2	3.8	10.8	78.1	79.5
2	50	96.75	3.25	25.9	76.7	75.0
3	100	96.2	3.8	48.2	71.9	65.4

a HPLC data measured at 210 nm.

The tan color of the crude isolated product (Figure S4) suggested that a charcoal treatment may be effective in improving product color and eliminating inorganic impurities. To test this hypothesis, a 10 g sample of crude **2** (taken from Table 8, entry 3) was dissolved in EtOAc (10 V) and treated with 20 wt % activated charcoal at 80 °C for 3 h. After filtration through Celite and solvent removal under a vacuum, an off-white solid was obtained with an assay purity of 94.2%, which indicates that this method could be used to obtain material of sufficient purity for subsequent chemistry.

## Conclusions

The important aniline intermediate **2** for the in-development TB treatment of sutezolid has been synthesized using a low-cost, proof-of-concept synthetic route that puts sulfide in a dual role as a nucleophilic reagent for thiomorpholine ring closure and as a reductant in a Zinin reaction. After optimization, each of the three steps in this new route was demonstrated at scales up to 100 g, with combined yields for the process ranging from 53 to 68%. The two new structural building blocks for this route, sodium sulfide and diethanolamine (DEA), are inexpensive commodity chemicals that replace thiomorpholine, the cost-driving fragment in previous sutezolid preparative routes. By using additional sulfide as a reductant for the nitro functionality, a separate stoichiometric or catalytic transition metal reduction of a nitrobenzene intermediate has also been avoided, which should provide additional cost savings. The modest purity of the product in the final step can be corrected by a simple charcoal treatment. However, we intend to explore further reactions in this process as part of a larger route redesign project,^22^ and one focus of that work will be to determine the suitability of subsequent intermediates as purge points for rejecting impurities.

## Experimental Section

Reagents and solvents were purchased from Aldrich Chemical Co., Fisher Scientific, Alfa Aesar, Acros Organics, Oakwood, or TCI. Liquid reagents were purified by distillation when necessary. Unless otherwise noted, solid reagents were used without further purification. Methylene chloride (DCM) and dimethylformamide (DMF) were taken from a solid-sorbent solvent dispensing system purchased from Pure Process Technologies or distilled as described in the literature. For all compounds, ^1^H and ^13^C NMR spectra were recorded on a Bruker Avance III 600 MHz spectrometer. Chemical shifts were measured relative to the residual solvent resonance for ^1^H and ^13^C NMR (CDCl_3_ = 7.26 ppm for ^1^H and 77.0 ppm for ^13^C, DMSO-*d*_6_ = 2.50 ppm for ^1^H and 39.5 ppm for ^13^C, CD_3_OD = 3.31 ppm for ^1^H and 49.0 ppm for ^13^C, and D_2_O = 4.79 ppm for ^1^H). Coupling constants (*J*) are reported in hertz (Hz). The following abbreviations were used to designate signal multiplicity: s, singlet; d, doublet; t, triplet; q, quartet, p, pentet; dd, doublet of doublet; ddd, doublet of doublet of doublet; dt, double of triplet; ddt, doublet of doublet of triplet; m, multiplet; br, broad. Reactions were monitored by HPLC using the methods described in the Supporting Information. Yields and purities were determined using HPLC by reference to independently prepared samples of the compounds of interest. Glassware was oven-dried at 120 °C, assembled while hot, and cooled to ambient temperature under an inert atmosphere. Reference samples of compounds **1**, **2, 8**, and **10** where prepared according to the literature reports.^17,26^

### Preparation of 2-[(2-Fluoro-4-nitrophenyl)(2-hydroxyethyl)amino]ethanol (**4**)

A 1 L, three-neck round-bottom flask equipped with an overhead stirrer and internal temperature probe was charged with diethanolamine (198.26 g, 1.89 mol, 3.0 equiv). The mixture was heated to 90 °C (internal temperature) with stirring (200 rpm). A color change from colorless to light yellow may be observed as the reagent is heated. After achieving constant temperature, 3,4-difluoronitrobenzene (100.0 g, 69.6 mL, 628.57 mmol, 1 equiv) was added at a rate of 50 mL/h using a syringe pump. (Note: This reaction is highly exothermic; therefore, controlled addition is required. Ideally, the addition rate would be adjusted such that the internal reaction temperature does not increase by more than ∼5 °C.) As this reagent was added, the reaction mass turned to a deep red color. After complete addition, the mixture was further stirred for a total of 2 h of reaction time. At this point, an HPLC IPC of the crude material showed complete consumption of the starting material. The heating was stopped, and water (500 mL, 5 V) was added slowly to the reaction mixture, which dropped the internal temperature from 90 to ∼50 °C. The reaction mass was allowed to cool to 30 °C over the course of approximately 1.5 h, and it was further cooled to an internal temperature 0–5 °C using an ice bath and stirred for additional 2 h. The solids were isolated by vacuum filtration through Buchner funnel and washed with ice-cold water (200 mL × 2) and then with MTBE (200 mL). The bright-yellow solid was transferred to a vacuum oven and dried overnight at 50 °C (16 h) to afford the product in 94.8% yield (149.48 g), with 97.4% assay purity and 96.8 LCAP. ^1^H NMR (600 MHz, CD_3_OD): δ 7.89 (ddd, *J* = 17.4, 12.1, 2.3 Hz, 2H), 7.03 (t, *J* = 9.2 Hz, 1H), 3.77 (t, *J* = 5.8 Hz, 4H), 3.68 (t, *J* = 5.6 Hz, 4H); ^13^C NMR (150 MHz, CD_3_OD): δ 151.7 (d, *J* = 243.9 Hz), 144.8 (d, *J* = 6.9 Hz), 138.7 (d, *J* = 8.5 Hz), 122.2 (d, *J* = 1.9 Hz), 116.9 (d, *J* = 4.9 Hz), 113.9 (d, *J* = 28.1 Hz), 60.9 (d, *J* = 2.1 Hz), 56.5 (d, *J* = 5.4 Hz); ^19^F NMR (565 MHz, CD_3_OD): δ −124.3 ppm. These data match those previously reported.^20^

### Preparation of 1-{(2-Fluoro-4-nitrophenyl)[2-(mesyloxy)ethyl]amino}-2-(mesyloxy)ethane (**5**)

The diol derivative **4** (100.0 g, 398.82 mmol, 1.0 equiv), acetonitrile (500 mL; 5 V), and triethylamine (138.8 mL, 997.04 mmol, 2.5 equiv) were charged into a 2 L, three-neck round-bottom flask equipped with an overhead stirrer, internal temperature probe, and nitrogen gas inlet. The reaction mixture was stirred (250 rpm) as it was cooled to an internal temperature of 0–5 °C using an ice-salt bath. Then, mesyl chloride (77.2 mL, 997.04 mmol, 2.5 equiv) was added dropwise while maintaining an internal temperature below 15 °C, and this mixture was allowed to stir for a total of 1 h. The temperature was allowed to warm up to 20 °C (room temperature) for an additional 4 h of total reaction time. At this time, an HPLC IPC showed full consumption of starting diol derivative. Water (400 mL, 4 V) was added, and the reaction mass was stirred overnight (16 h) at room temperature (20 °C). The reaction mass was cooled to 0–5 °C using an ice bath and stirred for an additional 2 h. The solids were isolated by vacuum filtration through Buchner funnel, washed with ice-cold water (200 mL × 2) and then with MTBE (200 mL), and dried under vacuum oven overnight (16 h) at 50 °C to afford a yellow solid in 89.8% yield (144.50 g), with 99.2% assay purity and 98.4 LCAP. ^1^H NMR (600 MHz, DMSO-*d*_6_): δ 8.01 (dd, *J* = 14.6, 2.7 Hz, 1H), 7.96 (dd, *J* = 9.2, 2.7 Hz, 1H), 7.20 (t, *J* = 9.3 Hz, 1H), 4.37 (t, *J* = 5.5 Hz, 4H), 3.86 (t, *J* = 5.4 Hz, 4H), 3.15 (s, 6H); ^13^C NMR (150 MHz, DMSO-*d*_6_): δ 150.3 (d, *J* = 244.9 Hz), 142.6 (d, *J* = 6.8 Hz), 138.0 (d, *J* = 8.9 Hz), 121.2, 117.2 (d, *J* = 4.4 Hz), 113.0 (d, *J* = 27.7 Hz), 67.5 (2C), 51.0 (d, *J* = 5.5 Hz, 2C), 36.7 (2C); ^19^F NMR (565 MHz, DMSO-*d*_6_): δ −121.4 ppm. HRMS (ESI/QTOF) *m*/*z*: [M + Na]^+^ Calcd for C_12_H_17_FN_2_NaO_8_S_2_ 423.0308, found 423.0310.

### Preparation of 3-Fluoro-4-(1,4-thiazinan-4-yl)aniline (**2**)

A 2 L, three-neck round-bottom flask equipped with an overhead stirrer and internal temperature probe was charged with EtOH:H_2_O (750 mL, 7.5 V), dimesyl compound **5** (100 g, 249.8 mmol, 1.0 equiv), and sodium sulfite (15.74 g, 124.9 mmol, 0.5 equiv). This mixture was heated to 70 °C (internal temperature) with stirring (250 rpm). Once the reaction temperature was achieved, a solution of Na_2_S·9H_2_O (72 g, 300 mmol, 1.2 equiv) in EtOH:H_2_O (250 mL, 2.5 V) was added dropwise with an Eldex pump over 30 min. After 1 h of reaction time, the remaining Na_2_S·9H_2_O (114 g, 480 mmol, 1.8 equiv) was added in three lots (addition interval of ∼20 min). The reaction mixture maintained at temperature for 4 more hours to complete the reaction. Starting material consumption was confirmed by an HPLC IPC. At this point, the pH of the reaction was adjusted to 11 by 1 M HCl (aq., 3 V ≈300 mL). The reaction mixture was filtered through a Buchner funnel to remove inorganic materials, and the solids were rinsed with ^*i*^PrOAc (2× 1 V, 200 mL). The filtrate was extracted with ^*i*^PrOAc (2× 4 V, 400 mL). The combined organic layers were concentrated under reduced pressure and dried under high-vacuum conditions to afford 48.20 g of faint yellow solid with 71.9% assay purity (65.4% assay-corrected yield).

The purity of this crude product could be enhanced by a charcoal treatment, as described here. A 10 g sample of the crude product and a magnetic stirrer were added into a round-bottom flask along with EtOAc (100 mL, 10 V). Activated charcoal (2 g, 20 mol % by weight, pH = 6–9) was added, and the reaction mass was heated to 80 °C for 3 h. This mixture was then filtered through a pad of Celite and washed with EtOAc (3 × 50 mL). The combined filtrates were concentrated under reduced pressure and dried under high vacuum to obtain an off-white solid product (7.72 g, 96.2% qNMR purity, and 94.2% assay purity). ^1^H NMR (600 MHz, DMSO-*d*_6_): δ 6.78 (dd, *J* = 9.9, 8.4 Hz, 1H), 6.33–6.28 (m, 2H), 5.02 (s, 2H), 3.04–3.03 (m, 4H), 2.70–2.68 (m, 4H); ^13^C NMR (150 MHz, DMSO-*d*_6_): δ 156.6 (d, *J*_C–F_ = 242.2 Hz), 145.9 (d, *J*_C–F_ = 11.0 Hz), 130.1 (d, *J*_C–F_ = 10.0 Hz), 122.1 (d, *J*_C–F_ = 4.4 Hz), 109.6 (d, *J*_C–F_ = 2.5 Hz), 101.6 (d, *J*_C–F_ = 23.2 Hz), 53.9, 27.7; ^19^F NMR (565 MHz, DMSO-*d*_6_): δ −123.9 ppm. HRMS (ESI/QTOF) *m*/*z*: [M + Na]^+^ Calcd for C_10_H_13_FN_2_NaS 235.0681, found 235.0680. Data matched with those previously reported.^17^

## Supporting Information Available

Characterization data for impurity **6**, pictures of the reaction mixtures and products for each step, NMR spectra, and analytical methods (PDF)
