# Supplementary material for: A Tandem Ring Closure and Nitrobenzene Reduction with Sulfide Provides an Improved Route to an Important Intermediate for the Anti-Tuberculosis Drug Candidate Sutezolid
Source: Org Process Res Dev. 2024 Mar 27;28(4):1195–204. doi: 10.1021/acs.oprd.4c00014 (PMC11036509; doi:10.1021/acs.oprd.4c00014)
Supplement: Supplementary file 1 — op4c00014_si_001.pdf [file op4c00014_si_001.pdf]

A Tandem Ring Closure and Nitrobenzene Reduction with Sulfide Provides an Improved Route to an Important Intermediate for the Anti-Tuberculosis Drug Candidate Sutezolid

Supporting Information

Hanuman P. Kalmode,[†] *Ongolu Ravikumar,*[†] *Dinesh J. Paymode,*[†] *John Bachert, Justina M. Burns, Rodger W. Stringham, Sarah L. Aleshire, Ryan C. Nelson**

Medicines for All Institute, 737 N 5th St., Box 980100, Richmond, Virginia 23298

† These authors contributed equally to the work presented here.

Contents:

Isolation and characterization of 2-{[2-(2-Fluoro-4-nitrophenoxy)ethyl](2-fluoro-4-nitrophenyl)amino}ethanol (6)……………………………………………………………2

Figure S1. Images of the SNAr Reaction between DFNB and DEA………………………….3

Figure S2. Images of the Mesylation Reaction of Compound 4……………………………….4

Figure S3. Images of the Reaction of Compound 5 with Sodium Sulfide…………………….5

Figure S4. Images of Compound 2 Before and After Charcoal Treatment………………….5

NMR Spectra………………………………...……………………………………………...6

Analytical Methods and HPLC Spectra………..…………………………………………18

Isolation and characterization of 2-{[2-(2-Fluoro-4-nitrophenoxy)ethyl](2-fluoro-4-nitrophenyl)amino}ethanol (6). This compound was isolated from a sample of impure S_NAr reaction product using silica gel column chromatography with a gradient of 5-10% ethyl acetate in hexane as the eluent. ^1H NMR (600 MHz, CD_3OD): δ 8.05 – 8.03 (m, 1H), 7.99 (dd, J = 10.9, 2.7 Hz, 1H), 7.92 (dd, J = 9.3, 2.7 Hz, 1H), 7.87 (dd, J = 14.9, 2.6 Hz, 1H), 7.26 (t, J = 8.7 Hz, 1H), 7.12 (t, J = 9.2 Hz, 1H), 4.42 (t, J = 5.3 Hz, 2H), 4.05 (t, J = 5.1 Hz, 2H), 3.80 (t, J = 5.8 Hz, 2H), 3.73 (t, J = 5.9 Hz, 2H); ^{13}C NMR (150 MHz, CD_3OD): δ 153.73 (d, J = 10.6 Hz), 153.05 (d, J = 71.7 Hz), 151.41 (d, J = 65.8 Hz), 144.59 (d, J = 6.9 Hz), 142.30 (d, J = 7.4 Hz), 139.32 (d, J = 8.7 Hz), 122.17 (d, J = 2.0 Hz), 122.04 (d, J = 3.4 Hz), 117.48 (d, J = 4.7 Hz), 114.65 (d, J = 1.5 Hz), 113.96 (d, J = 28.1 Hz), 112.92 (d, J = 23.1 Hz), 69.38 (d, J = 2.3 Hz), 60.62 (d, J = 1.4 Hz), 56.27 (d, J = 4.7 Hz), 53.16 (d, J = 6.5 Hz); ^{19}F NMR (565 MHz, CD_3OD): δ -123.7, -132.8 ppm. HRMS (ESI/QTOF) m/z: $[M + Na]^+$ Calculated for $C_{16}H_{15}F_2N_3NaO_6$ 406.0827, found 406.0829.

Figure S1. Images of the S_NAr Reaction between DFNB and DEA (Step 1)

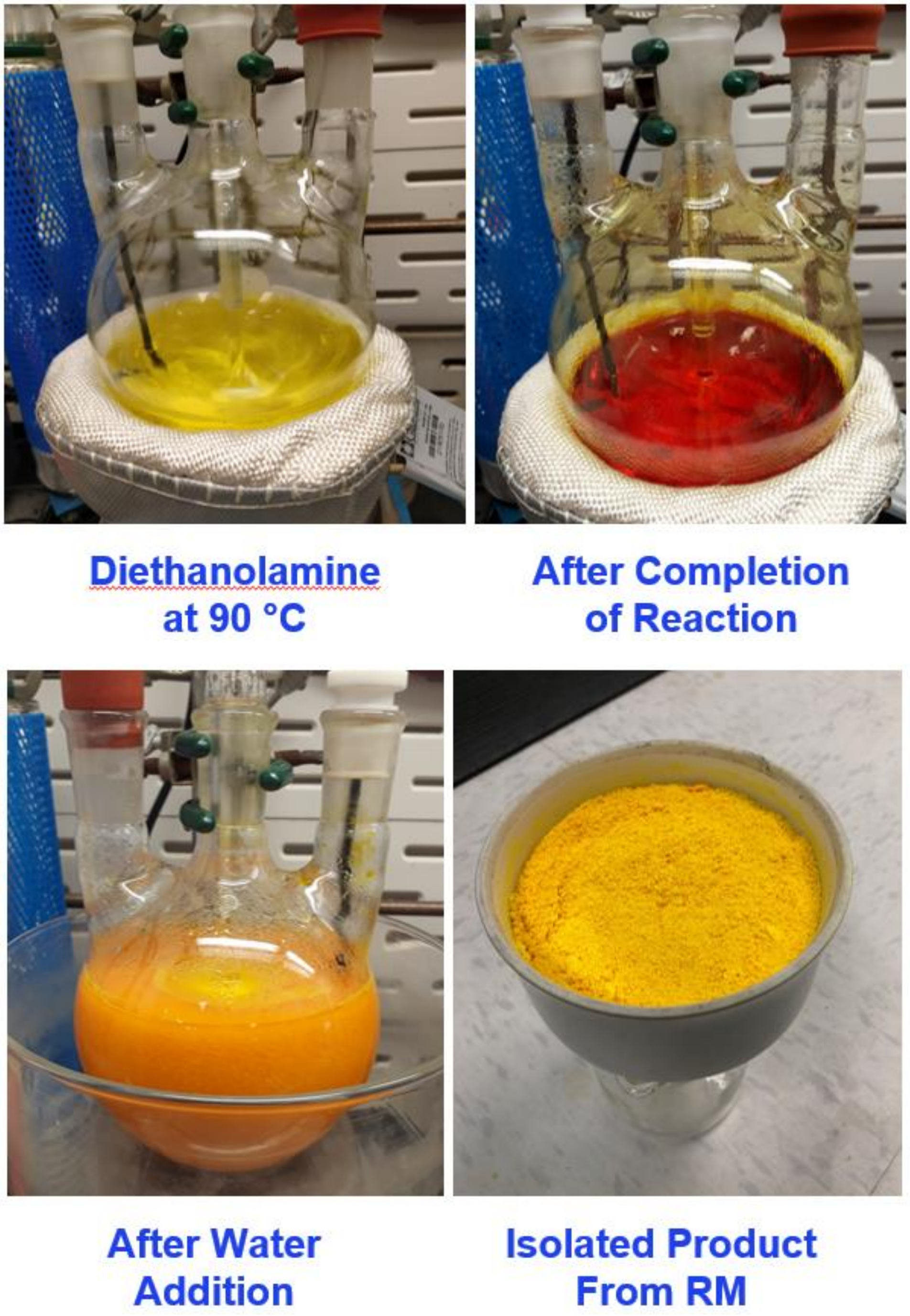

Figure S2. Images of the Mesylation Reaction of Compound 4

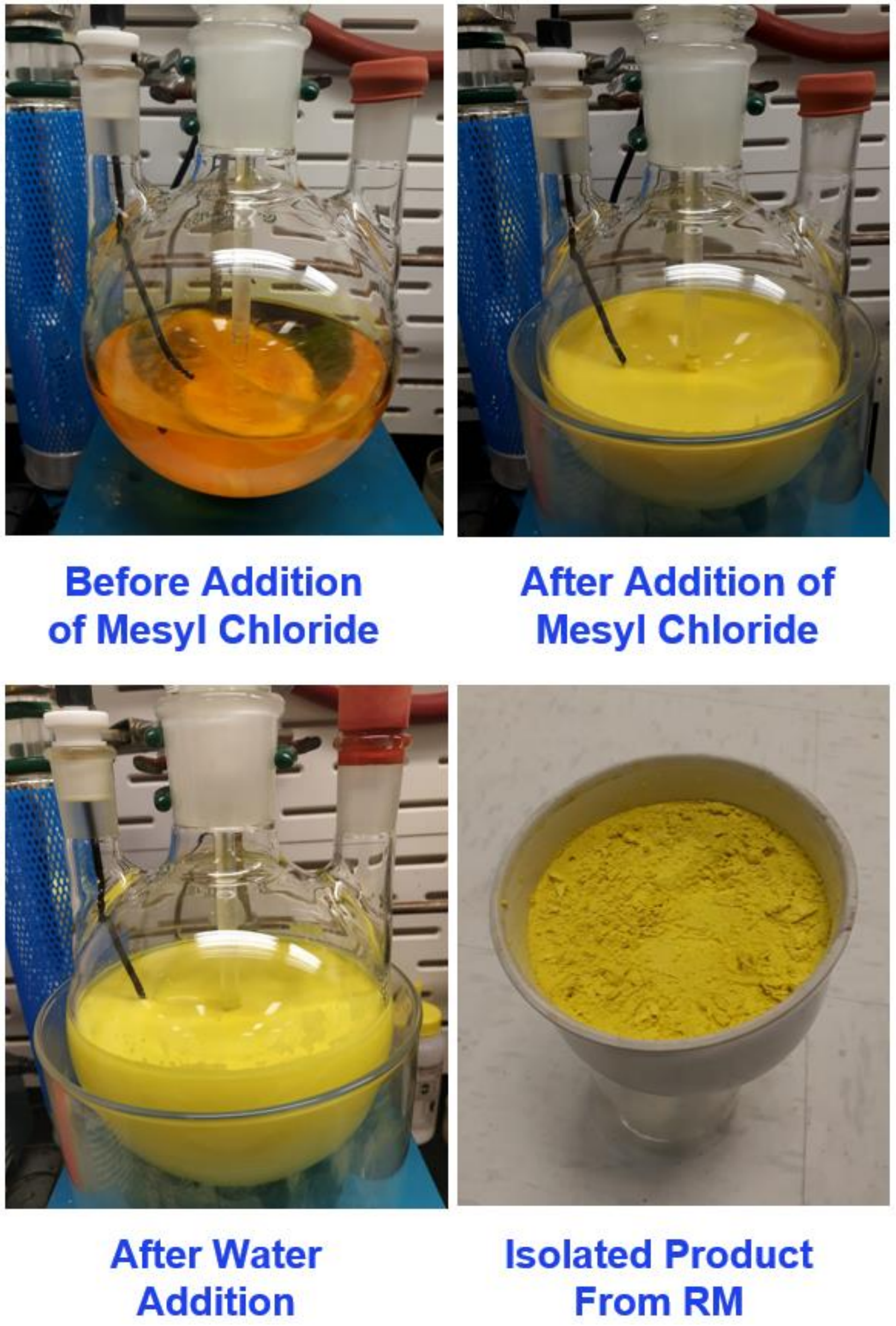

Figure S3. Images of the Reaction of Compound 5 with Sodium Sulfide

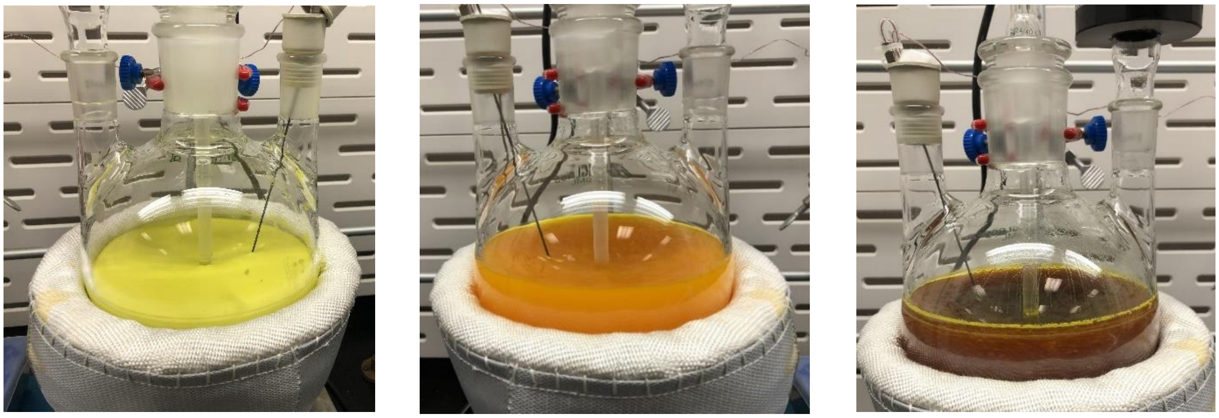

Start of the reaction After 1 hour After completion of the reaction

Figure S4. Images of Compound 2 Before and After Charcoal Treatment

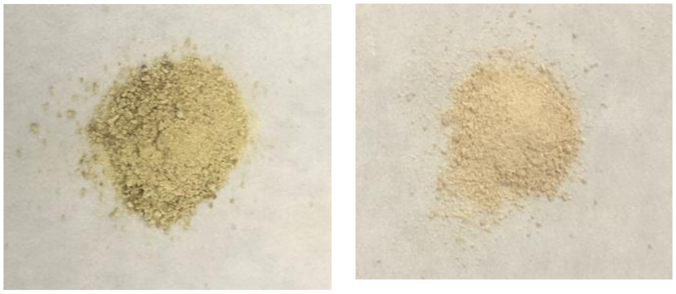

Before charcoal treatment After charcoal treatment

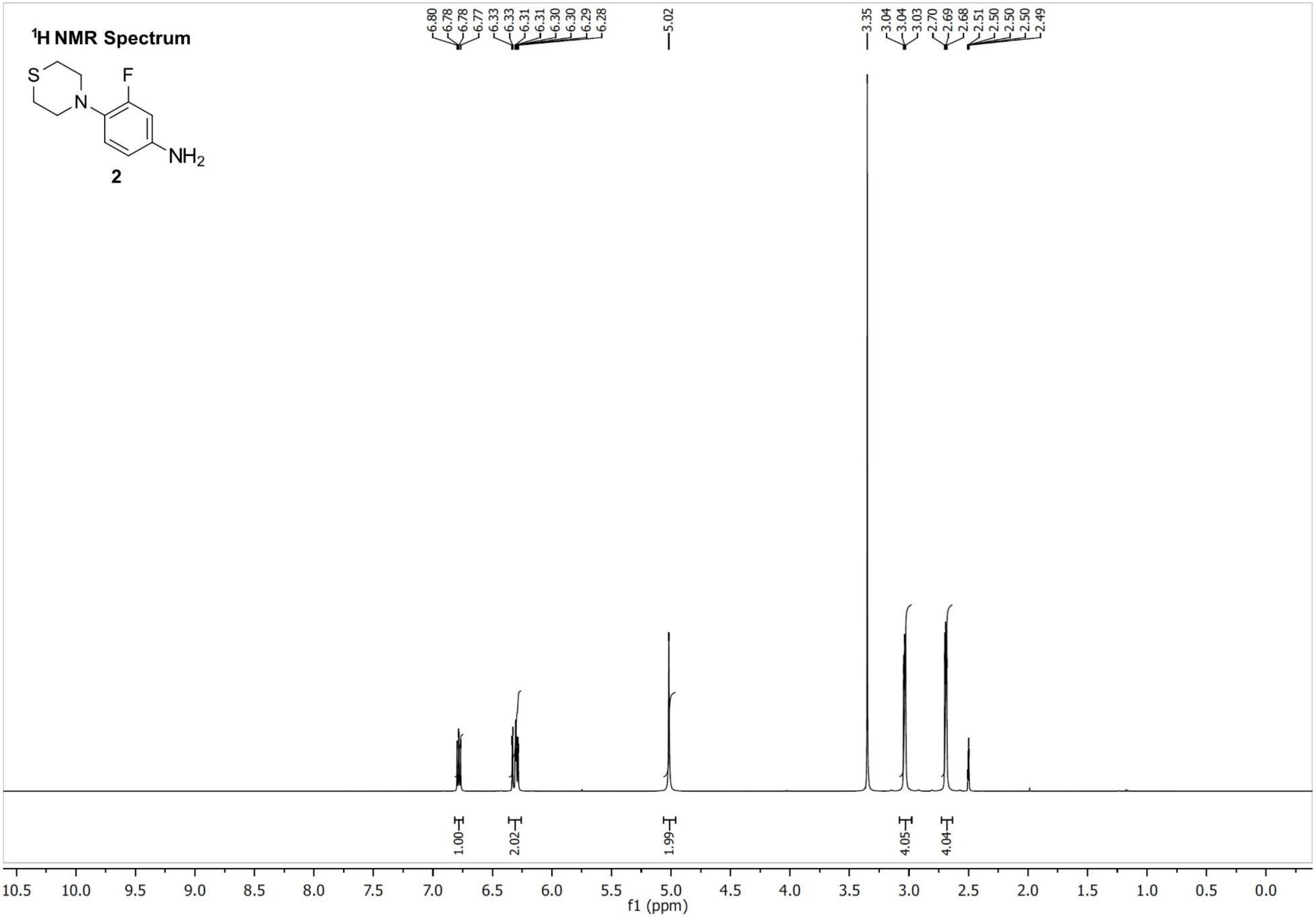

1H NMR Spectrum
S
N
F
NH2
2
6.80
6.78
6.78
6.77
6.33
6.33
6.31
6.31
6.30
6.30
6.29
6.28
5.02
3.35
3.04
3.04
3.03
2.70
2.69
2.68
2.51
2.50
2.50
2.50
2.49
1.00
2.02
1.99
4.05
4.04
10.5 10.0 9.5 9.0 8.5 8.0 7.5 7.0 6.5 6.0 5.5 5.0 4.5 4.0 3.5 3.0 2.5 2.0 1.5 1.0 0.5 0.0
f1 (ppm)

^{13}C NMR Spectrum

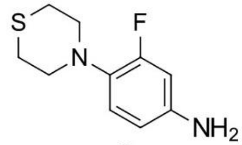

2

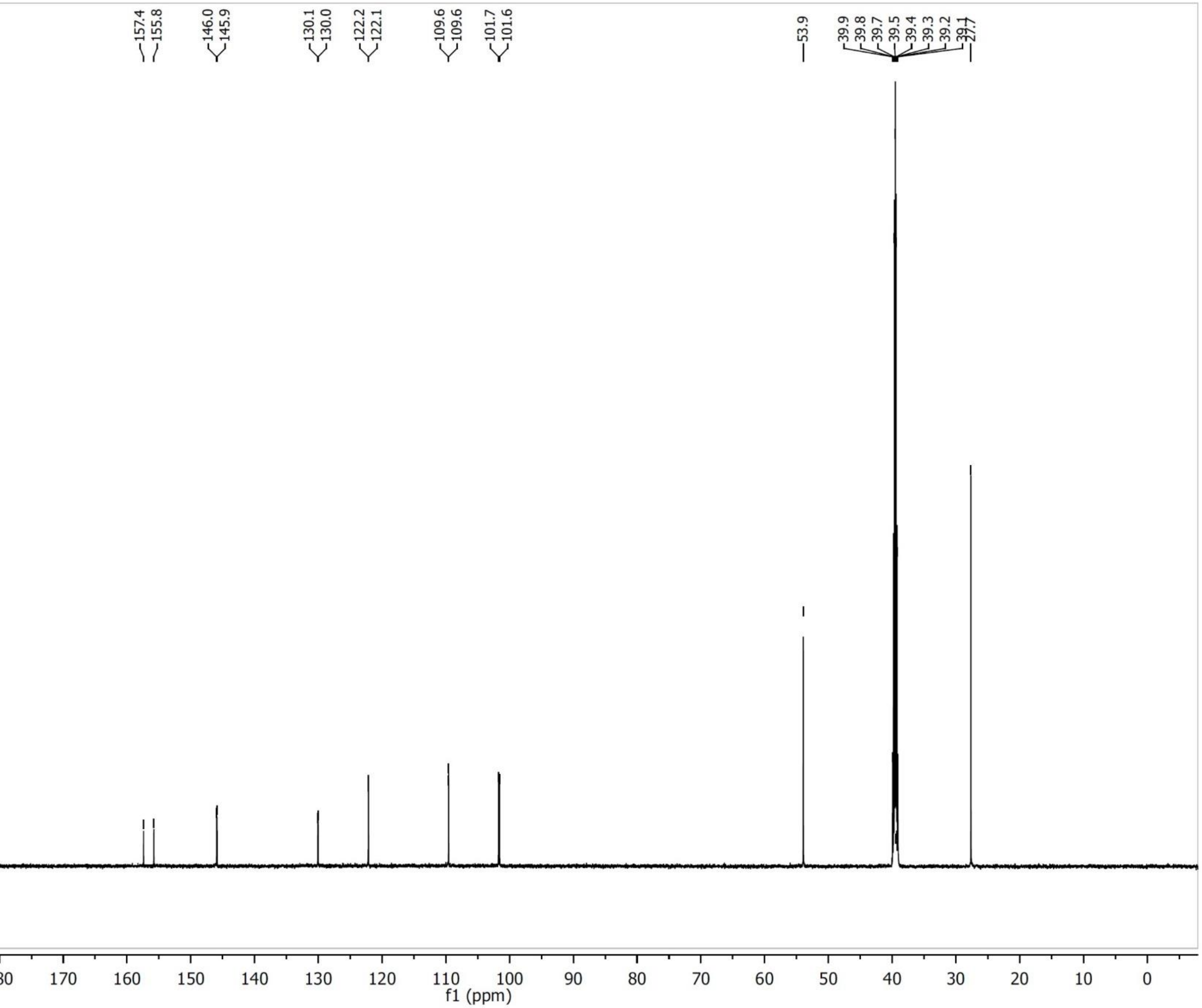

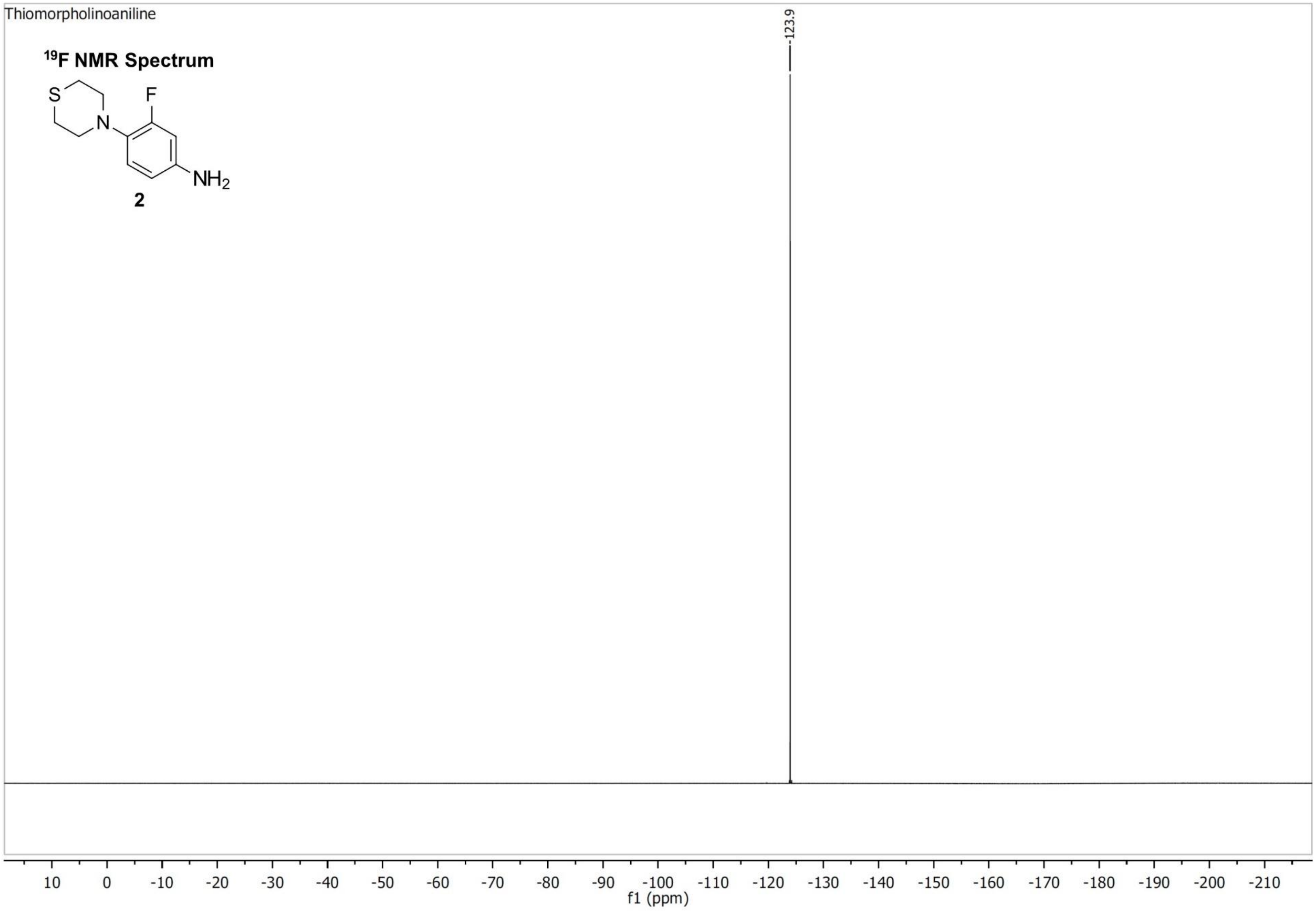
Thiomorpholinoaniline
19F NMR Spectrum
S
N
F
NH2
2
-123.9
10 0 -10 -20 -30 -40 -50 -60 -70 -80 -90 -100 -110 -120 -130 -140 -150 -160 -170 -180 -190 -200 -210
f1 (ppm)

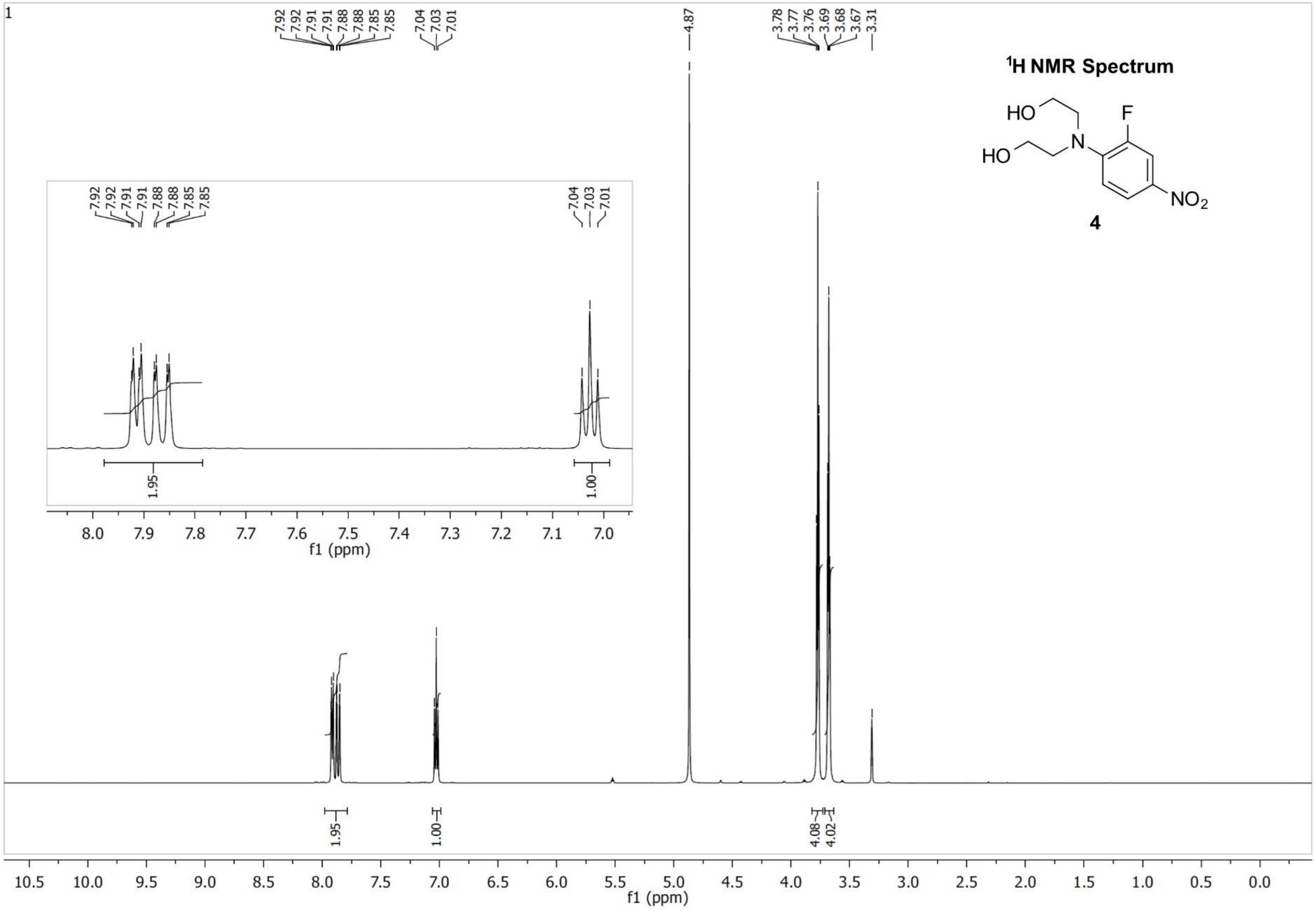
1
¹H NMR Spectrum
HO
HO
N
F
NO2
4
7.92
7.92
7.91
7.91
7.88
7.88
7.85
7.85
7.04
7.03
7.01
4.87
3.78
3.77
3.76
3.69
3.68
3.67
3.31
1.95
1.00
4.08
4.02
f1 (ppm)

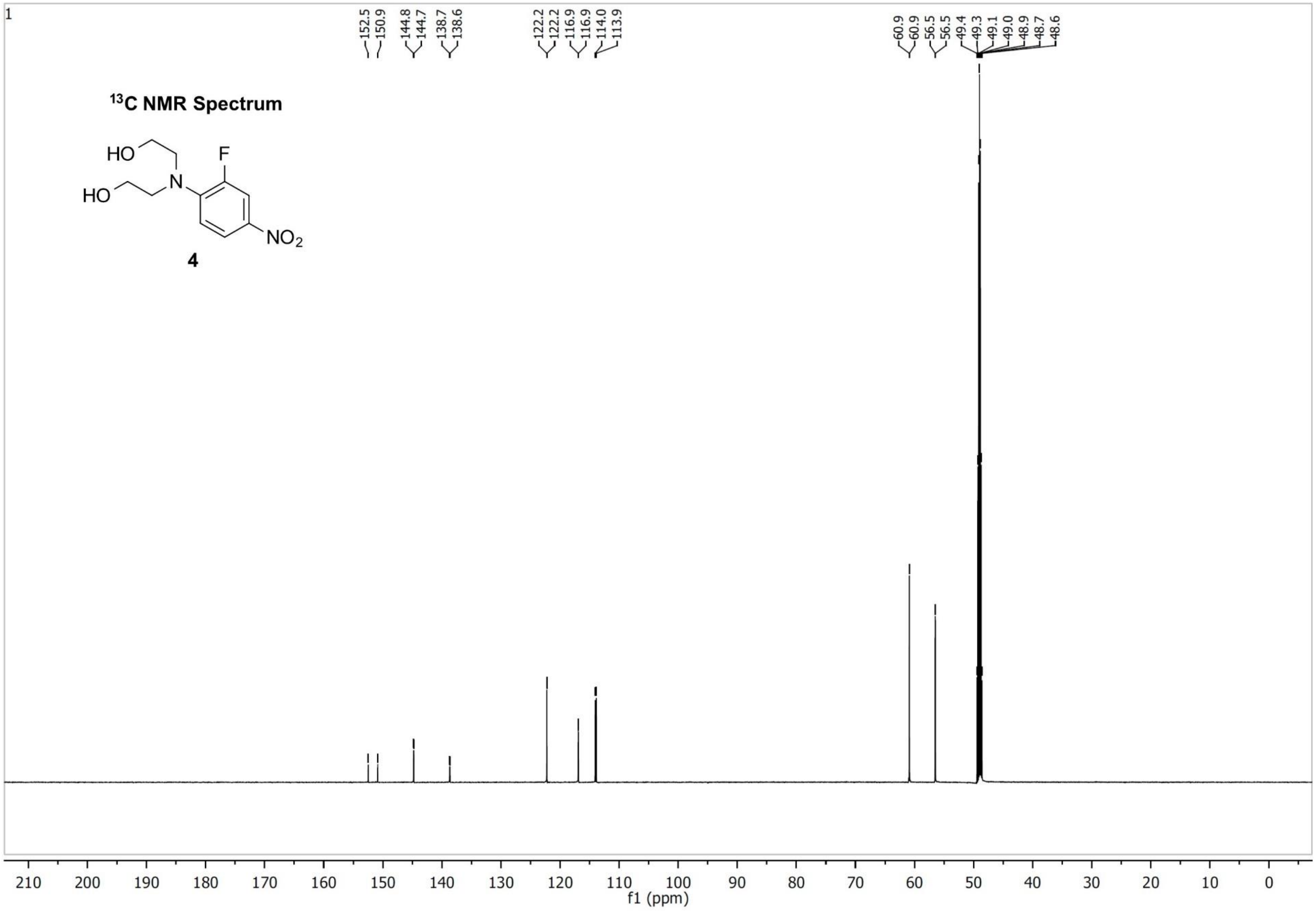

1
152.5
150.9
144.8
144.7
138.7
138.6
122.2
122.2
116.9
116.9
114.0
113.9
60.9
60.9
56.5
56.5
49.4
49.3
49.1
49.0
48.9
48.7
48.6
13C NMR Spectrum
HO
HO
N
F
NO2
4
210 200 190 180 170 160 150 140 130 120 110 100 90 80 70 60 50 40 30 20 10 0
f1 (ppm)

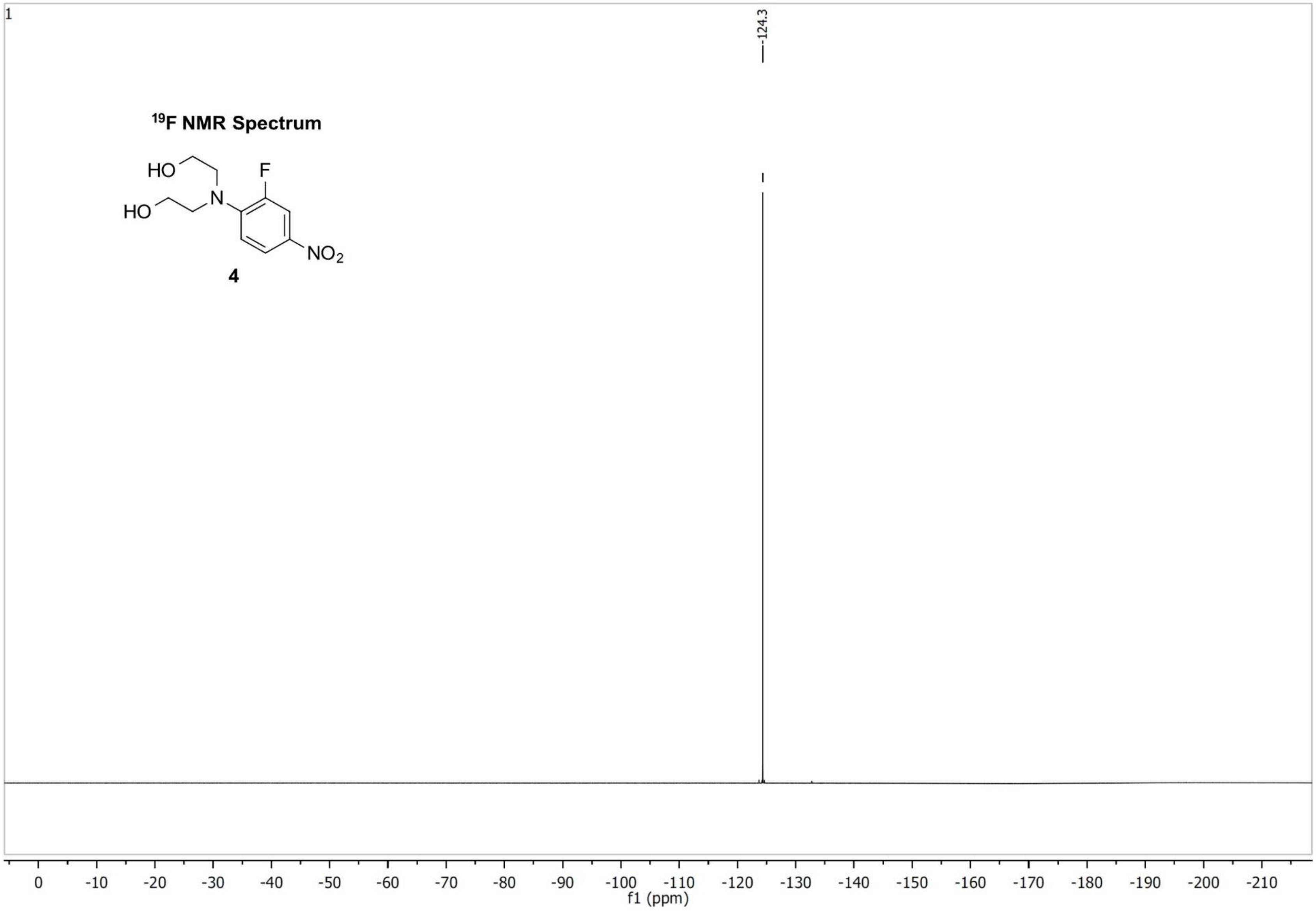
1
-124.3
19F NMR Spectrum
HO
HO
N
F
NO2
4
0 -10 -20 -30 -40 -50 -60 -70 -80 -90 -100 -110 -120 -130 -140 -150 -160 -170 -180 -190 -200 -210
f1 (ppm)

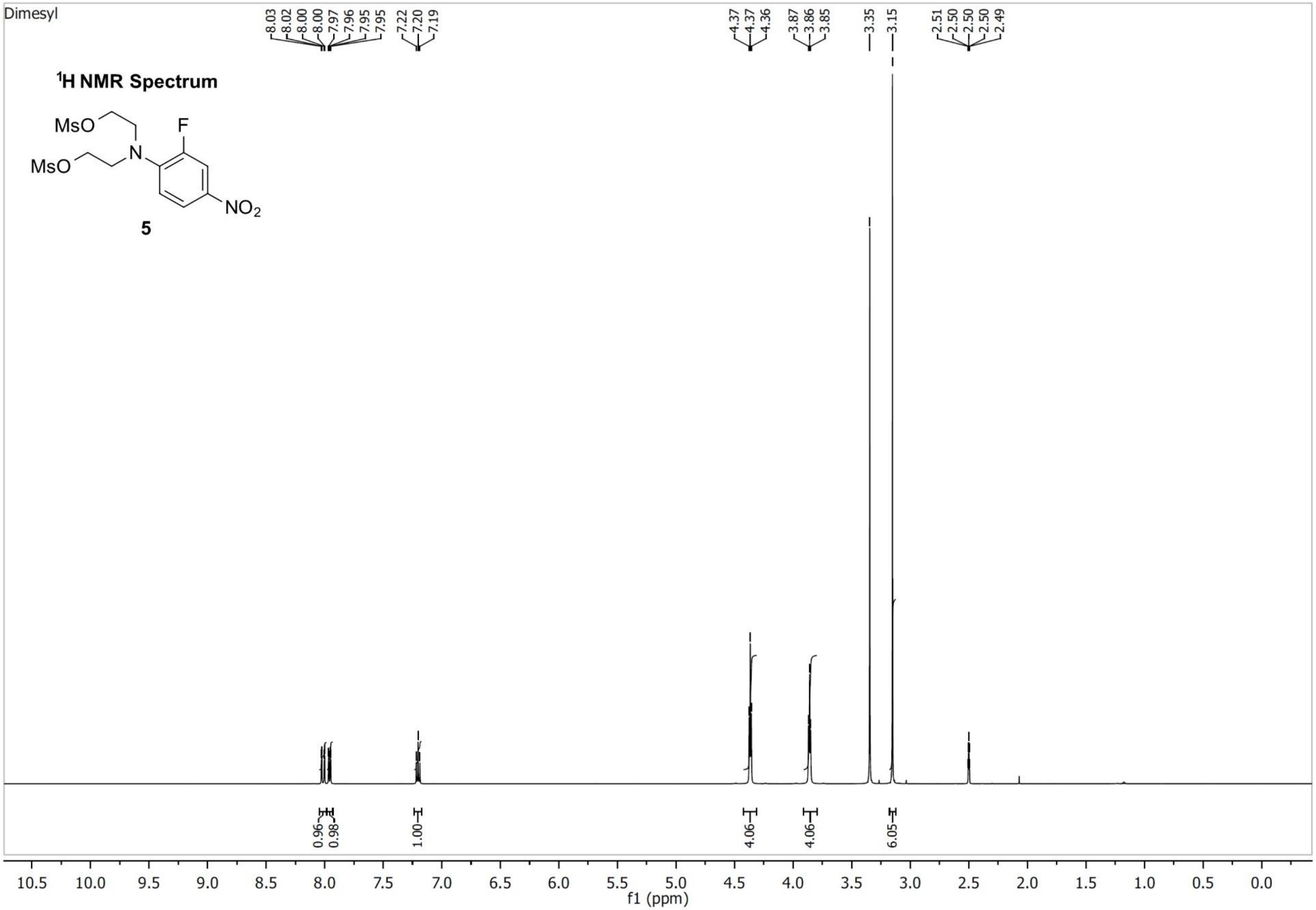
Dimesyl
¹H NMR Spectrum
MsO
MsO
F
N
NO2
5
8.03
8.02
8.00
8.00
7.97
7.96
7.95
7.95
7.22
7.20
7.19
4.37
4.37
4.36
3.87
3.86
3.85
3.35
3.15
2.51
2.50
2.50
2.50
2.49
0.96
0.98
1.00
4.06
4.06
6.05
10.5 10.0 9.5 9.0 8.5 8.0 7.5 7.0 6.5 6.0 5.5 5.0 4.5 4.0 3.5 3.0 2.5 2.0 1.5 1.0 0.5 0.0
f1 (ppm)

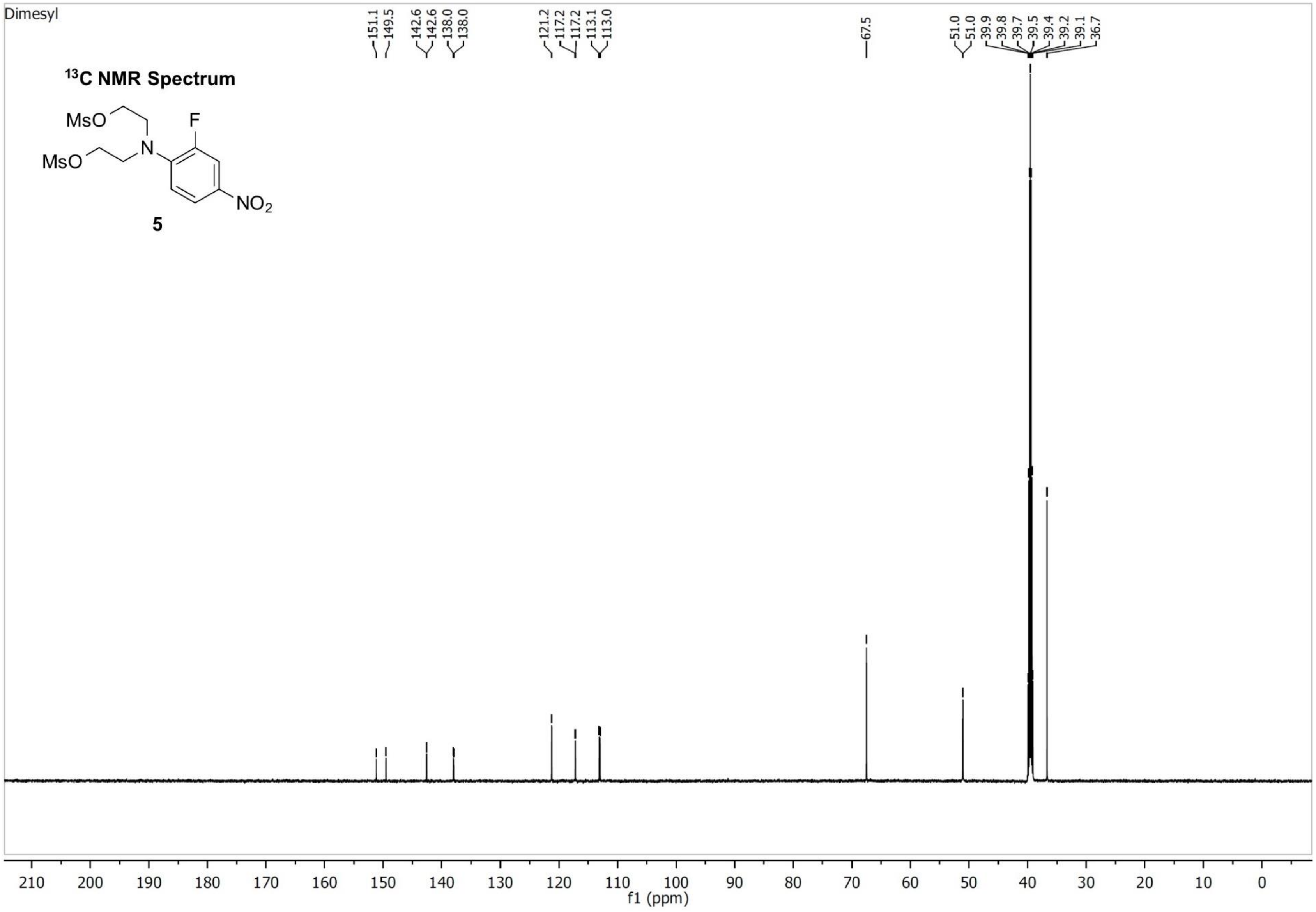
Dimesyl
13C NMR Spectrum
MsO
MsO
N
F
NO2
5
151.1
149.5
142.6
142.6
138.0
138.0
121.2
117.2
117.2
113.1
113.0
67.5
51.0
51.0
39.9
39.8
39.7
39.5
39.4
39.2
39.1
36.7
210 200 190 180 170 160 150 140 130 120 110 100 90 80 70 60 50 40 30 20 10 0
f1 (ppm)

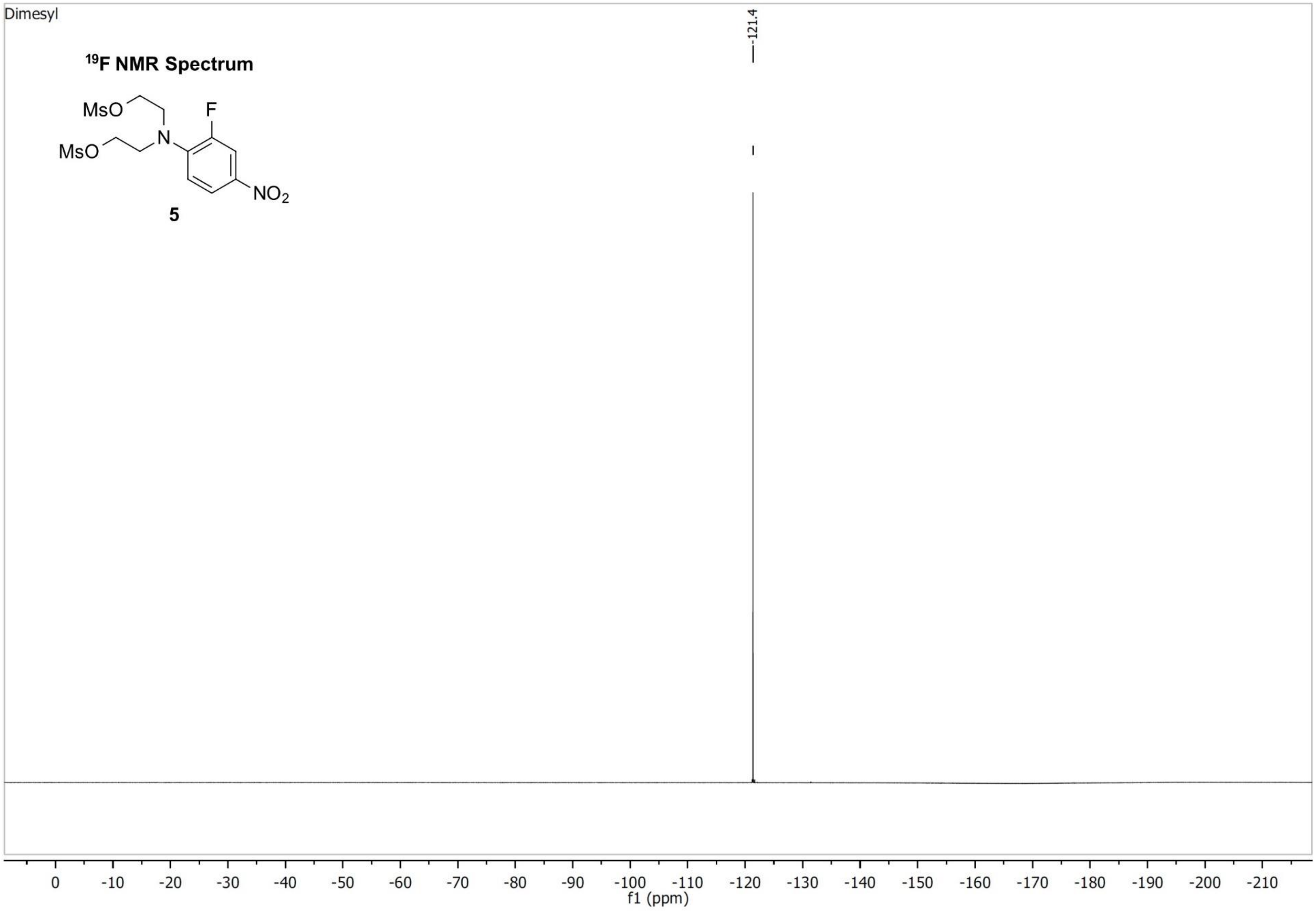
Dimesyl
19F NMR Spectrum
MsO
MsO
N
F
NO2
5
-121.4
0
-10
-20
-30
-40
-50
-60
-70
-80
-90
-100
-110
-120
-130
-140
-150
-160
-170
-180
-190
-200
-210
f1 (ppm)

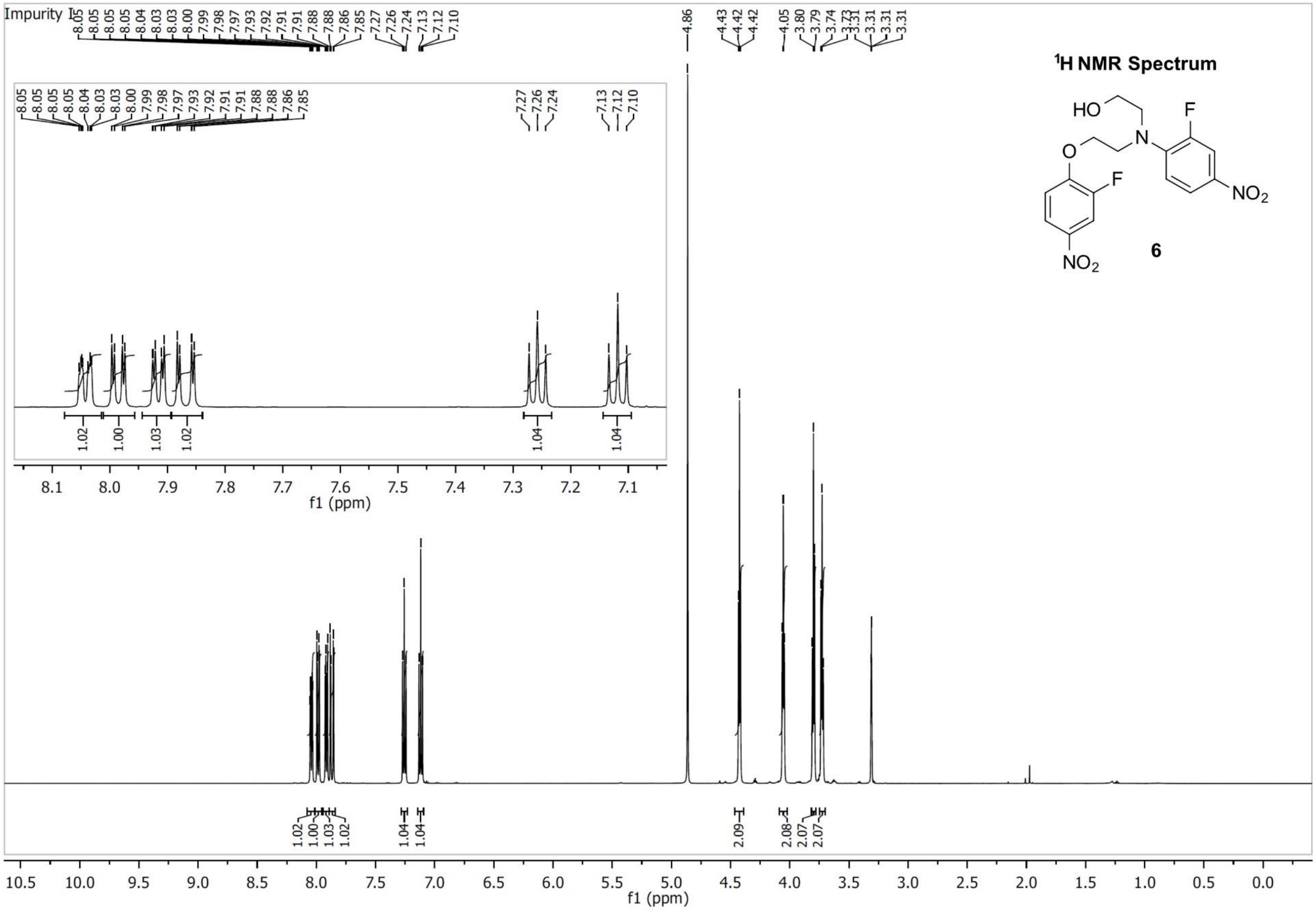

Impurity I
1H NMR Spectrum
HO
F
O
N
F
NO2
NO2
6
8.05 8.05 8.05 8.05 8.04 8.03 8.03 8.00 7.99 7.98 7.97 7.93 7.92 7.91 7.91 7.88 7.88 7.86 7.85
7.27 7.26 7.24
7.13 7.12 7.10
4.86
4.43 4.42 4.42
4.05 3.80 3.79 3.74 3.73 3.31 3.31 3.31 3.31
1.02 1.00 1.03 1.02
1.04
1.04
2.09
2.08 2.07 2.07
8.1 8.0 7.9 7.8 7.7 7.6 7.5 7.4 7.3 7.2 7.1
f1 (ppm)
10.5 10.0 9.5 9.0 8.5 8.0 7.5 7.0 6.5 6.0 5.5 5.0 4.5 4.0 3.5 3.0 2.5 2.0 1.5 1.0 0.5 0.0
f1 (ppm)

Impurity I

^{13}C NMR Spectrum

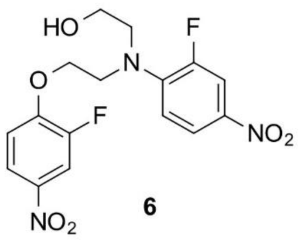

6

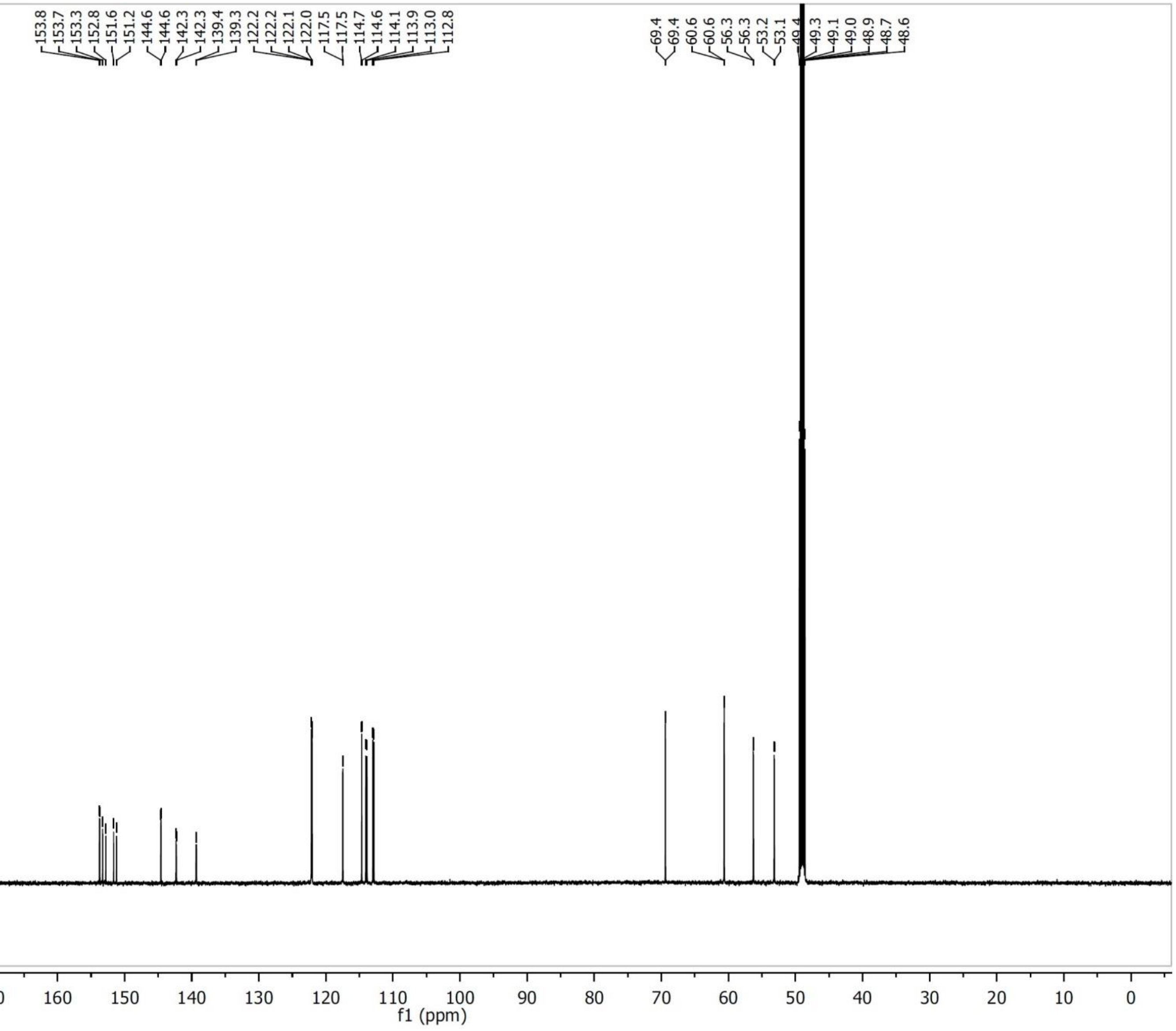

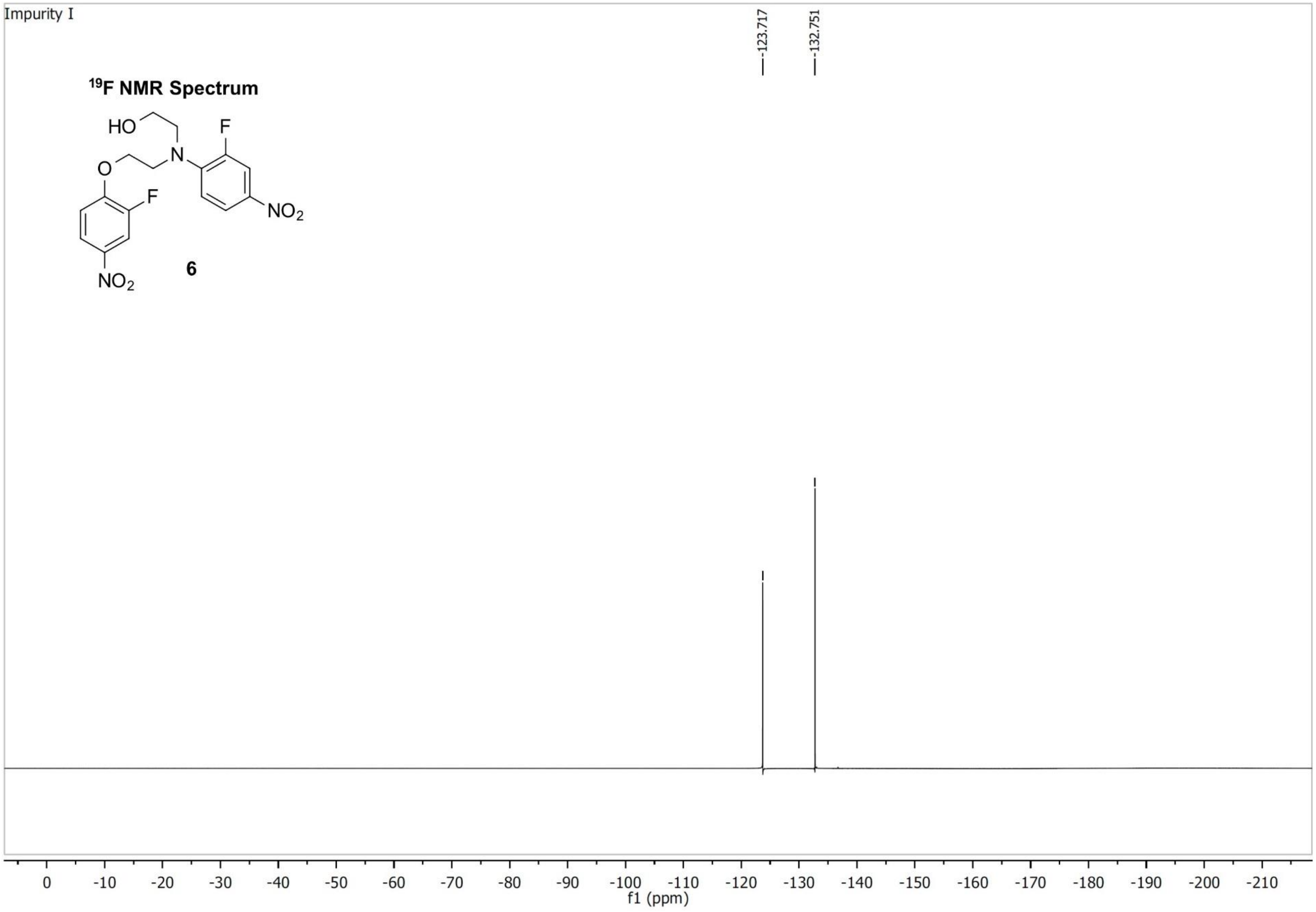
Impurity I
-123.717
-132.751
19F NMR Spectrum
HO
F
O
N
F
NO2
NO2
6
0
-10
-20
-30
-40
-50
-60
-70
-80
-90
-100
-110
-120
-130
-140
-150
-160
-170
-180
-190
-200
-210
f1 (ppm)

Analytical Methods and HPLC Chromatograms

Conditions:

Column: Agilent Eclipse XDB 4.6 x 250 mm; 5 µm

Mobile Phase A: 0.1% H_3PO_4

Mobile Phase B: Acetonitrile

Injection volume: 1 µL

Column temp: 30 C

Flow rate: 1.5 mL/min

Detector wavelength(s): 210 nm and 380 nm

* Compounds 1, 4, and 5 can be measured at 380 nm. Others should be measured at 210 nm

LC Gradient Table:

Time (min)	%A	%B
0.0	80%	20%
17.0	30%	70%

Post-run equilibration: 4.0 min

Sample preparation:

Prepare samples at approximately 1 mg/mL in acetonitrile

Retention Times

Compound	Time (min)	Relative RF (mg/mL)*	Relative RF (M)*
**DEA** (Diethanolamine)	1.5	Not determined	
**Compound 2**	3.1		
**Compound 4**	5.7		
**Compound 5**	10.7		
**DFNB** (Difluoro nitrobenzene)	11.6		
**Compound 1**	15.6		

Representative Chromatogram(s)

210 nm

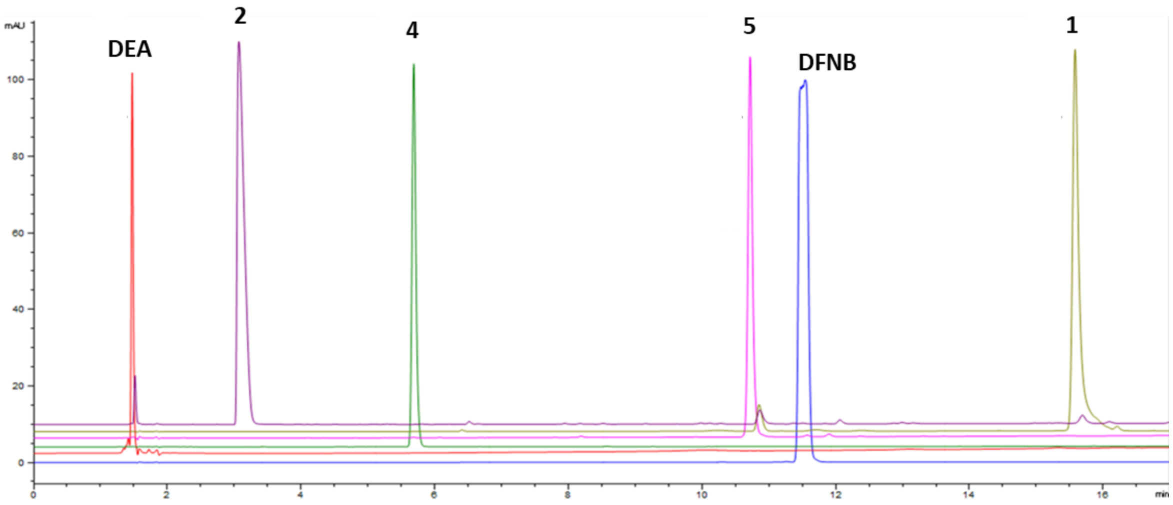

380 nm

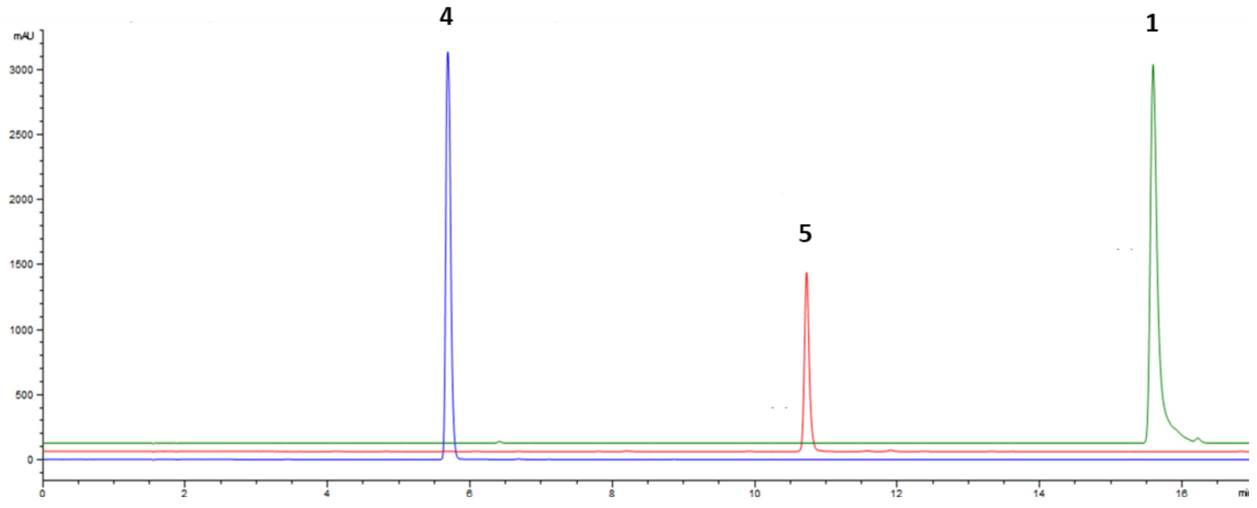

UV-Vis spectrum of **DFNB**

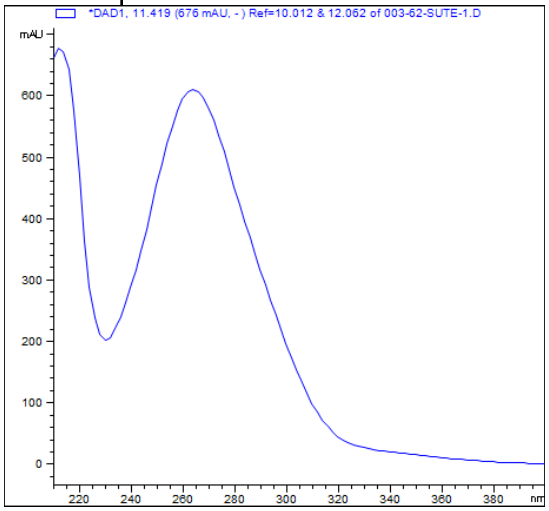

UV-Vis spectrum of **DEA**

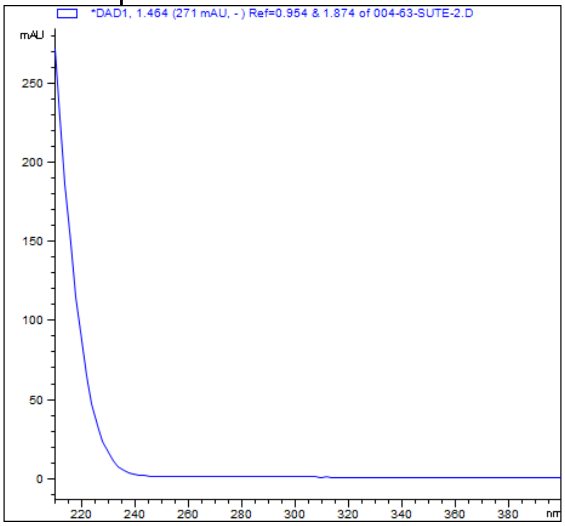

UV-Vis spectrum of **4**

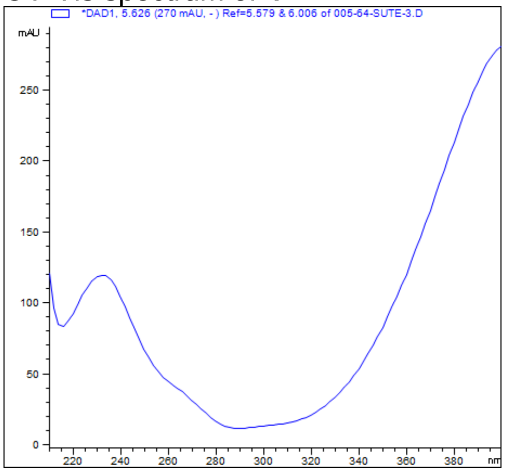

UV-Vis spectrum of **5**

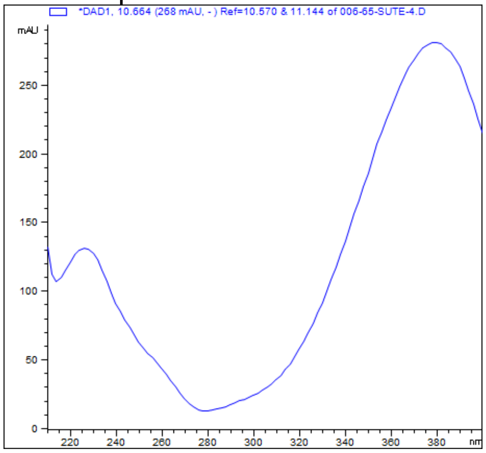

UV-Vis spectrum of **1**	UV-Vis spectrum of **2**
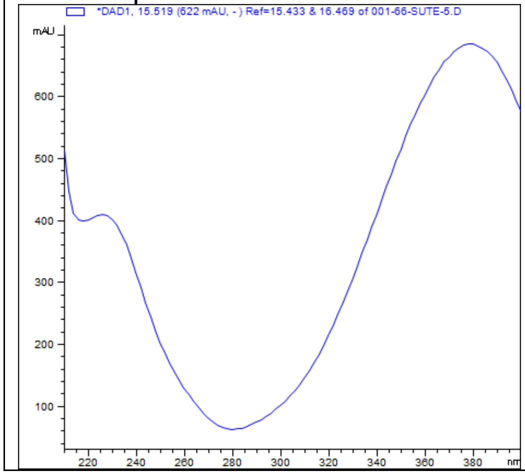	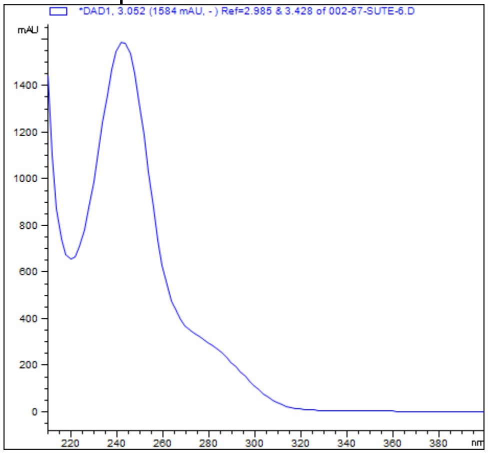
